# Variance constraints strongly influenced model performance in growth mixture modeling: a simulation and empirical study

**DOI:** 10.1186/s12874-020-01154-0

**Published:** 2020-11-12

**Authors:** Jitske J. Sijbrandij, Tialda Hoekstra, Josué Almansa, Margot Peeters, Ute Bültmann, Sijmen A. Reijneveld

**Affiliations:** 1 Department of Health Sciences, Community and Occupational Medicine Groningen, University Medical Center Groningen, University of Groningen, Groningen, The Netherlands; 2 Department of Interdisciplinary Social Science, Utrecht University, Utrecht, Netherlands

**Keywords:** Simulation studies, Longitudinal studies, Developmental trajectories, Growth mixture model, Variance misspecification, Model selection

## Abstract

**Background:**

Growth Mixture Modeling (GMM) is commonly used to group individuals on their development over time, but convergence issues and impossible values are common. This can result in unreliable model estimates. Constraining variance parameters across classes or over time can solve these issues, but can also seriously bias estimates if variances differ. We aimed to determine which variance parameters can best be constrained in Growth Mixture Modeling.

**Methods:**

To identify the variance constraints that lead to the best performance for different sample sizes, we conducted a simulation study and next verified our results with the TRacking Adolescent Individuals’ Lives Survey (TRAILS) cohort.

**Results:**

If variance parameters differed across classes and over time, fitting a model without constraints led to the best results. No constrained model consistently performed well. However, the model that constrained the random effect variance and residual variances across classes consistently performed very poorly. For a small sample size (*N* = 100) all models showed issues. In TRAILS, the same model showed substantially different results from the other models and performed poorly in terms of model fit.

**Conclusions:**

If possible, a Growth Mixture Model should be fit without any constraints on variance parameters. If not, we recommend to try different variance specifications and to not solely rely on the default model, which constrains random effect variances and residual variances across classes. The variance structure must always be reported Researchers should carefully follow the GRoLTS-Checklist when analyzing and reporting trajectory analyses.

**Supplementary Information:**

The online version contains supplementary material available at 10.1186/s12874-020-01154-0.

## Background

Growth Mixture Modeling (GMM) has become a standard statistical approach in grouping people from heterogeneous populations based on their development over time (e.g. mental health during adolescence [1, 2] or antisocial behavior [3]). Several simulation studies have shown that GMM outperforms other statistical methods, such as latent class analysis and latent class growth analysis [4–6]. However, GMMSs are complex models, i.e. many different parameters are estimated: mean trend of trajectories, class sizes, residual variances over time and across classes and random effects across classes. Recently, the Guidelines for Reporting on Latent Trajectory Studies (GRoLTS-Checklist) have been published, with criteria for reporting the complexity of latent trajectory analyses [7]. When the most unrestricted GMM is estimated, convergence issues may arise, especially for smaller sample sizes (e.g. inadmissible values such as negative variances) [1, 8] Convergence issues are common in GMM, and if the models do not properly converge or yield to inadmissible values, their results are not reliable.

Convergence issues regarding variances can be addressed by limiting the number of estimated variance parameters, i.e. by constraining certain parameters as being equal over time or across classes. But when these variance parameters are unequal, this can lead to considerable bias in the entire model. For instance, constraining residual variances to be equal over time leads to lower classification accuracy and less frequent detection of the correct number of classes [9, 10]. The same holds for constraining residual variance across classes [5] and of random effects across classes [10–12].

Difference in variances across trajectory classes or over time are a common scenario in empirical applications. For example, Moffit coined the developmental taxonomy of classes of antisocial behavior [13]. In Moffit’s taxonomy, classes can differ in the level of antisocial behavior and in the variability of this behavior. One class, labeled as abstainers, may show no behavioral problems with almost no within-class variation [3]. A second class, the adolescent-limited group, may show a decline in behavioral problems over time, with large variability in early adolescence but more homogeneity in late adolescence. A third class, labeled as the life-course persistent group, may show consistently high antisocial behavior with constant variability over time.

Even though the GRoLTS -Checklist emphasizes the importance of reporting variance constraints, these are rarely reported in the literature [7, 14], which hinders transparency and replicability. For instance, none of the 38 papers which were included in the GRoLTS review reported on constraints of variance parameters. The authors of the GRoLTS Checklist warned that “researchers should be aware that findings might be altered if the variance–covariance matrix is redefined”. The lack of reported details regarding variance structure and convergence issues related to variance parameters makes us suspect that many researchers apply the simpler default GMM settings in their software, without considering the alternatives. To the best of our knowledge, no study has assessed which parameter constraints are least likely to induce bias. Thus, to date, it is unclear which type of variance constraints leads to the best outcomes [5, 10, 15].

The aim of this paper is to determine which variance parameters can best be constrained in GMMs of different samples sizes for best recovery of simulated classes, least biased estimates, and most accurate class assignment. This may help researchers to estimate a GMM, when convergence issues arise, which is as close to the population values as possible.

## Methods

We conducted a simulation study and verified the results with an empirical example. In this example, we used data on aggressive behavior from the TRacking Adolescent Individuals’ Lives Survey (TRAILS) cohort.

### Simulation study

#### GMM, random effects and residual variances

**Fig. 1:**
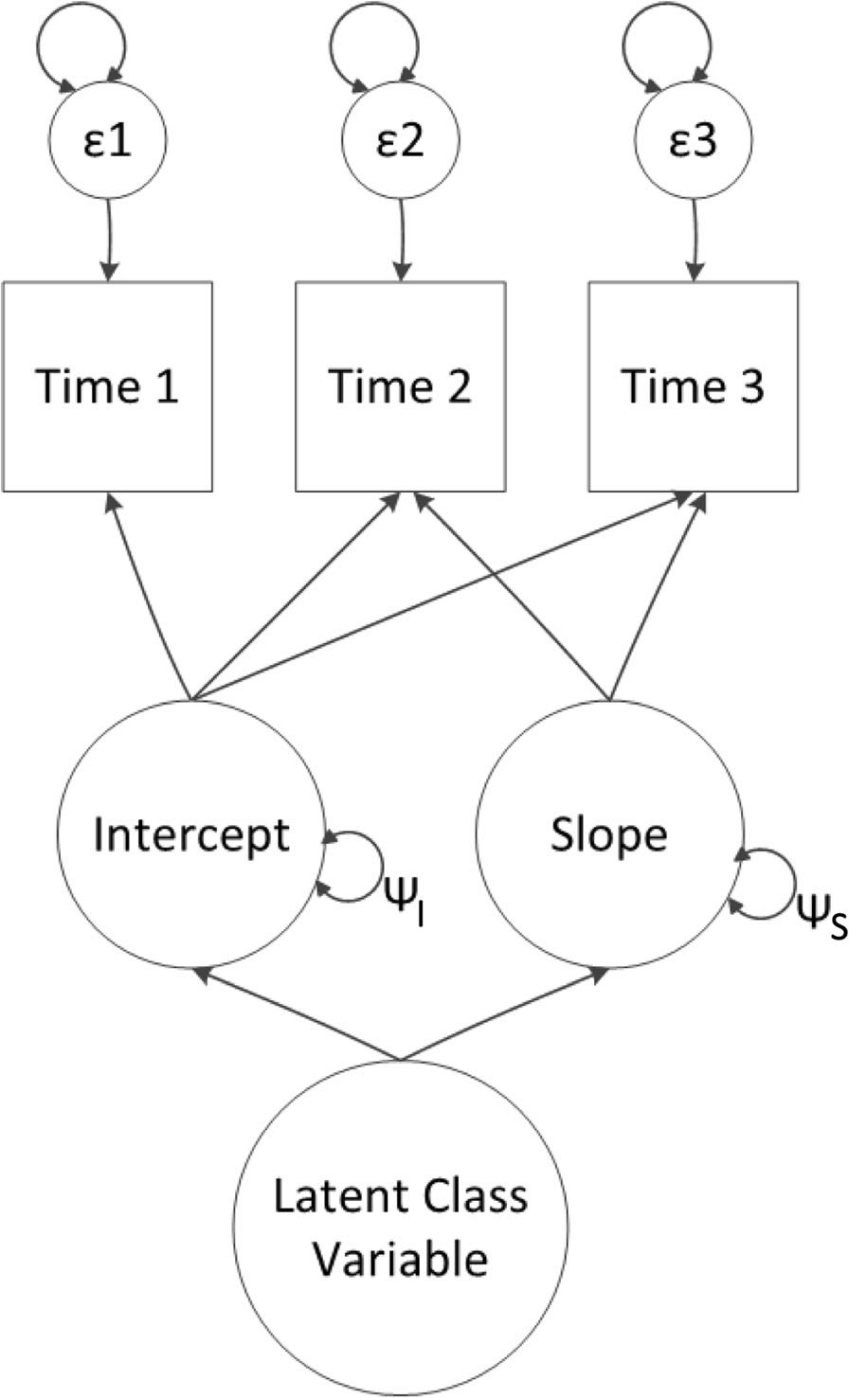
Diagram of Growth Mixture Modeling. The circular arrows above the epsilons represent the residual variances (which can be let free/constrained over time and over classes). The circular arrows accompanied with the letter psi represent the random effects (which can be let free/constrained over classes)

GMM (Fig. 1) is an extension of latent curve modeling (also known as growth curve modeling) that does not require the assumption that all individuals stem from one population [16]. GMM allows to identify unobserved classes of individuals based on their development over time, estimated by means of an intercept and slopes parameters. GMM includes random effects (i.e. the variation around that mean trajectory within the classes) and residual variances (i.e. the variance of the difference between the observed and estimate value for each individual at each time point). Comprehensive introductions to GMM are available (e.g. [7]).

#### Data generation

The population values in the simulation study were based on a review of GMM studies [3]. Our largest sample size was between the median and first quartile of the findings in the review (1000 individuals). We added two smaller sample sizes, since convergence issues are most common for smaller samples (100 and 300, both close to the minimum values in the review). The number of time points (5) corresponds to the median value in this review. The degree of separation between classes (intercepts of adjacent classes were either 1 (low separation) or 2 (high separation) standard deviations apart), based on the three studies identified in the review in which this was reported. The number of classes (3) was based on the first quartile of the reported number of classes in the review [3] (Supplementary Figure 1). See further https://osf.io/68ptf/?view_only=4e3bd94405b3427498449208d02a7b88 for all R code used to simulate and analyze the data.

We based the specification of the variance parameters on two empirical studies, by Kooken et al. (2018) and Morin et al. (2011) [1, 17], since variance parameters are rarely reported in GMM studies [7, 14]. We varied the size of residual variances over time and across classes, and the size of random effects over classes, resulting in eight different scenarios. In the least extreme scenario, the ratio of all parameters was 1:2:3 and in the most extreme scenario, the ratio was 1:5:10 (see Table 1). As an example: In Scenario 2, Class 3 had three times larger standard deviations of the residual variances and three times larger random effects than Class 1. Moreover, for all classes, the standard deviations were ten times larger at T5 than at T1.

**Table 1:** Parameter specification in the data generation of the scenarios with low separation between classes in the simulation study

Scenario		Class 1		Class 2		Class 3	
		Mean	Standard deviation	Mean	Standard deviation	Mean	Standard deviation
1	Intercept	3.00	0.3	4.00	0.60	5.00	0.90
	Slope	0.00	0.1	−0.30	0.20	0.30	0.30
	Residual variance T1, T2		0.25		0.50		0.75
	Residual variance T3		0.5		1.00		1.50
	Residual variance T4, T5		0.5		1.00		1.50
2	Intercept	3.00	0.3	4.00	0.60	5.00	0.90
	Slope	0.00	0.1	−0.30	0.20	0.30	0.30
	Residual variance T1, T2		0.1		0.20		0.30
	Residual variance T3		0.5		1.00		1.50
	Residual variance T4, T5		0.5		1.00		1.50
3	Intercept	3.00	0.12	4.00	0.60	5.00	1.20
	Slope	0.00	0.04	−0.30	0.20	0.30	0.40
	Residual variance T1, T2		0.25		0.50		0.75
	Residual variance T3		0.5		1.00		1.50
	Residual variance T4, T5		0.5		1.00		1.50
4	Intercept	3.00	0.12	4.00	0.60	5.00	1.20
	Slope	0.00	0.04	−0.30	0.20	0.30	0.40
	Residual variance T1, T2		0.1		0.20		0.30
	Residual variance T3		0.5		1.00		1.50
	Residual variance T4, T5		0.5		1.00		1.50
5	Intercept	3.00	0.3	4.00	0.60	5.00	0.90
	Slope	0.00	0.1	−0.30	0.20	0.30	0.30
	Residual variance T1, T2		0.1		0.50		1.00
	Residual variance T3		0.2		1.00		2.00
	Residual variance T4, T5		0.2		1.00		2.00
6	Intercept	3.00	0.12	4.00	0.60	5.00	1.20
	Slope	0.00	0.04	−0.30	0.20	0.30	0.40
	Residual variance T1, T2		0.10		0.50		1.00
	Residual variance T3		0.20		1.00		2.00
	Residual variance T4, T5		0.20		1.00		2.00
7	Intercept	3.00	0.30	4	0.60	5.00	0.90
	Slope	0.00	010	−.3	0.20	0.30	0.30
	Residual variance T1, T2		0.04		0.20		0.40
	Residual variance T3		0.20		1.00		2.00
	Residual variance T4, T5		0.20		1.00		2.00
8	Intercept	3.00	0.12	4	0.60	5.00	1.20
	Slope	0.00	0.04	−.03	0.20	0.30	0.40
	Residual variance T1, T2		0.04		0.20		0.40
	Residual variance T3		0.20		1.00		2.00
	Residual variance T4, T5		0.20		1.00		2.00

#### Evaluation criteria

We evaluated three criteria: (1) class recovery, (2) relative bias and (3) classification accuracy. Class recovery concerned whether the simulated and estimated classes could be linked. Non-recovery means that most individuals are identified as being dispersed across several estimated classes, whereas they were simulated as belonging to one class. Class recovery was calculated as the proportion of replications in which simulated and estimated classes could be linked. Relative bias concerned the difference between the simulated and estimated parameters, divided by the simulated parameter (population value) and was calculated for the intercept and for the slope parameters, for all three classes. For the intercept of the first class, the bias was not relative, since the simulated parameter value was 0. Classification accuracy concerns the percentage of individuals correctly assigned to a certain class (within those simulations where the original classes were recovered). It should be noted that in case of non-recovery, relative bias and classification accuracy could not be computed. As a secondary outcome, we reported the proportions of replications in which each model was found to be the most appropriate model based on the BIC, AIC, aBIC and entropy. The BIC, AIC and aBIC are commonly used to assess the best fitting models in GMM. Previous studies have indicated that the BIC and aBIC were able to detect the correct GMM more often than the AIC [4, 5, 18], but all fit indices tend to identify models with a higher number of classes than present in the population as best fitting. The entropy does not indicate the fit of the model, but indicates how well classes are separated and how well individuals fit in their respective class Although entropy is not a formal model fit index, it is often used as a measure for fit and therefore we have reported the entropy as well.

#### Analytical procedure

The simulated data were analyzed with increasingly constrained GMMs, starting with the unconstrained model (Model 0). Thereafter, the models with one constraint were estimated: a model with residual variance constrained over time (1A), a model with residual variances constrained across classes (1B) and model with random effects constrained across classes (1C). We ended with models with two constraints: a model with residual variance constrained over time and across classes (2A), a model with random effects constrained and residual variance constrained over time (2B) and a model with random effects constrained and residual variances constrained across classes (2C). We calculated the BIC (Bayesian Information Criterion), aBIC (Sample-size Adjusted BIC), AIC (Akaike Information Criterion) and entropy to aid model selection. R 3.4.0 [19] was used to simulate the data and analyze the Mplus output, Mplus 8 [20] to fit the GMMs, and MplusAutomation [21] to communicate between R and Mplus.

### Verification of simulation results with empirical data: the TRacking adolescents’ individual lives survey

The influence of the specification of random effect variances and residual variances in GMM was verified with the Tracking Adolescents’ Individual Lives Survey (TRAILS) cohort. TRAILS is a population-based cohort in the Netherlands including young adolescents followed into early adulthood. Assessment took place every 2 to 3 years, from the age of 11.1 years (SD 0.56) in 2000 up to the age of 25.7 years (SD 0.60) in 2016 [22], and is still ongoing.

#### Problem definition

We chose aggressive behavior as trajectory variable as we aimed to investigate the effect of constraining certain parameters which differ over time or classes. Aggression is expected to decrease over time, as behavioral control of adolescents increases when they get older [23]. In line with previous research, we expected at least two groups to emerge: a group with low aggression at the first measurement, which decreases over time, and at least one other group, which starts with a higher level of aggression [24]. An additional trajectory with a higher initial aggression level might emerge (e.g. [24–26]). We expect smaller variance in the first group than in the latter group(s) and the residual variance to decrease over time (i.e. overall aggressive behavior and variation in aggressive behavior decrease over time [24]).

#### Measures

Aggressive behavior is a syndrome scale measured by 17 items of the Youth Self-report (YSR) [27] in the first three measurement waves, and 15 items in the Adult Self-report (ASR) [28] in the next three measurement waves. The response categories were 0 (not true), 1 (somewhat or sometimes true) or 2 (very true or often true). We used mean values for all analyses.

#### Analytical procedure and model specification

The TRAILS data were analyzed with increasingly constrained models, starting with the unconstrained model and ending with the “classes-constrained model”. For each model, first a 1-class solution was fitted and the number of classes was increased until the model no longer converged properly or the fit indices indicated that the model no longer improved by adding an additional class. The model accounted for cubic growth over time. No random quadratic or cubic slopes were estimated, since these are rare in empirical data [14].

The final models were selected based on the BIC, aBIC and the Lo-Mendell-Rubin likelihood ratio test. Solutions were considered suboptimal if very small classes (i.e. < 5% of the sample) or very similar classes emerged. We followed the steps for analyzing GMMs according to the Ram & Grimm procedure and reported these according to the GRoLTS checklist, where possible [7, 29].

Since we were particularly interested in performance within smaller sample sizes, three subsamples of TRAILS of each 300 individuals were analyzed, to investigate the performance of the models for smaller samples. Only individuals with a maximum of one missing measurement point were sampled.

## Results

We consecutively present the results of the simulation study and of the empirical example.

### Simulation study

#### Overall results

For a sample size of 1000, the unconstrained model mostly outperformed all other models, but for smaller sample sizes this was not the always the case. Irrespective of sample size, the model which constrained residual variance and random effect variance across classes (Model 2C, from now on referred to as ‘classes-constrained model’) performed very poorly in terms of all evaluation criteria.

#### Sample size of 1000 and large separation between classes

**Fig. 2:**
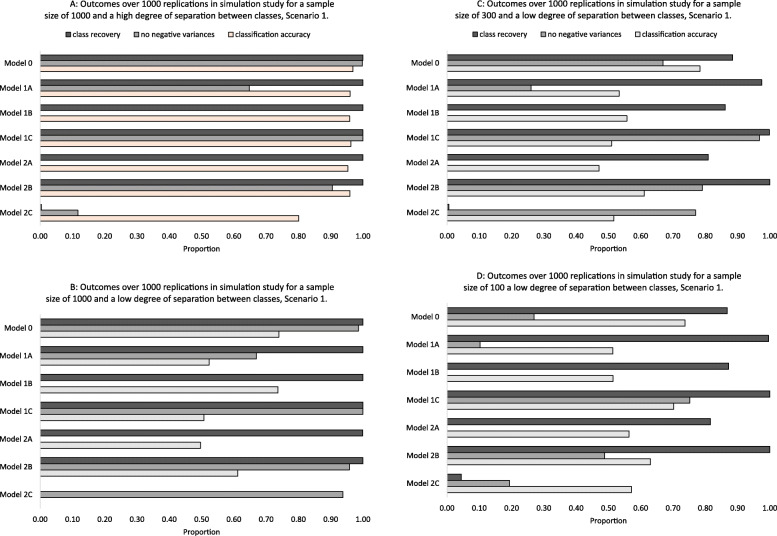
**a** Outcomes over 1000 replications in simulation study for a sample size of 1000 and a high degree of separation between classes, Scenario 1. **b** Outcomes over 1000 replications in simulation study for a sample size of 1000 and a low degree of separation between classes, Scenario 1. **c** Outcomes over 1000 replications in simulation study for a sample size of 300 and a low degree of separation between classes, Scenario 1. **d** Outcomes over 1000 replications in simulation study for a sample size of 100 a low degree of separation between classes, Scenario 1. Negative variances and classification accuracy are conditional on class recovery: Only if the simulated classes. Are recovered, the other two outcomes can be calculated. Class recovery: proportion of replications in which simulated and estimated classes could be linked, No negative variances: proportion of replications in which no negative variances occurred, classification accuracy: proportion of individuals correctly assigned to a certain class. A value of 1.00 indicates the best possible performance

For a sample size of 1000 and a high separation between classes, the unconstrained model performed best. All models performed well under all variance ratio scenarios except for the ‘classes-constrained model’ (Table 2, Supplementary Table 2 and Supplementary Table 3, illustrated for Scenario 1 in Fig. 2a). Negative variances occurred less often for the unconstrained model than for other models. The AIC, BIC and aBIC indicated that the unconstrained model was best fitting in all replications; the entropy was highest for the model which constrained residual variance over time and across classes (2A) and the “classes-constrained model” (Supplementary Table 1).

**Table 2:** Absolute values of relative bias of intercept (int) and slope per analysis model: findings over 1000 replications in the simulation for a sample size of 1000 and a high and low degree of separation between classes in Scenario 1

Model		Intercepts			Slopes		
		Class 1	Class 2	Class 3	Class 1	Class 2	Class 3
High separation and *N* = 1000							
0	Nothing constrained	.00	.00	.00	.00	.00	.00
1A	Residual variance time constrained	.00	.01	.00	.00	.02	.00
1B	Residual variance classes constrained	.00	.00	.00	.00	.00	.00
1C	Random effects constrained	.02	.02	.01	.00	.01	.02
2A	Residual variance constrained time and classes	.00	.00	.00	.00	.00	.00
2B	Random effects and residual variance time constrained	.00	.01	.00	.00	.04	.07
2C	Random effects and residual variance classes constrained	**.23**	**.17**	.01	.10	**.17**	**.29**
Low separation and N = 1000							
0	Nothing constrained	.00	.00	.00	.00	.01	.01
1A	Residual variance time constrained	.00	.01	.00	.00	.08	.08
1B	Residual variance classes constrained	.00	.01	.00	.00	.04	.00
1C	Random effects constrained	.01	.01	.02	.01	.01	.05
2A	Residual variance constrained time and classes	.00	.01	.00	.00	.06	.00
2B	Random effects and residual variance time constrained	.01	.01	.01	.01	.03	**.21**
2C	Random effects and residual variance classes constrained	-^a^	–	–	–	–	–

Relative bias: bias divided by true population value

Codes for relative bias: Bold ≥ .1

The variance ratio in Scenario 1 is 1:3 across classes and over time points, which means that the variance in the first class/time-point is three times smaller compared to the last class/time-point

^a^The bias could not be calculated, since the simulated classes were not recovered

#### Sample size of 1000 and small separation between classes

The unconstrained model performed best in terms of classification accuracy and relative bias (Fig. 2b). For a sample size of 1000 and a small separation between classes, class recovery was 100% for all models under all scenarios, except for the “classes-constrained model” (Fig. 2b, Supplementary Table 2). All models but the classes constrained model performed well in terms of relative bias. The BIC, aBIC and AIC indicated that the unconstrained model was the best fitting model, while the entropy was highest for model 2A and the” classes-constrained model” (Supplementary Table 1).

#### Sample size of 300 and small separation between classes

For a sample size of 300 and a small separation between classes, the unconstrained model performed best, except in Scenario 1 (Fig. 2c, Table 3, Supplementary Table 3C). In Scenario 1, no model performed best for all evaluation criteria: i.e. class recovery was highest in Models 1A, 1C and 2B, while classification accuracy was highest in the unconstrained model (Fig. 2c, Supplementary Table 2). In all scenarios, class recovery was close to 0% for the “classes-constrained model”. For the other models, class recovery ranged from 76.2–100%. In three scenarios (4, 7 and 8), classification accuracy was always highest for the unconstrained model and equally high for the model with random effects constrained (1C). In scenarios 2–8, the aBIC and AIC indicated the unconstrained model as best fitting in 96.8–100% of the replications (Supplementary Table 1). The BIC yielded similar results, except for Scenario 5.

**Table 3:** Absolute values of relative bias of intercept (int) and slope per analysis model: findings over 1000 replications in the simulation for a sample size of 300 and 100 and low degree of separation between classes in Scenario 1

Model		Intercept class 1	Intercept class 2	Intercept class 3	Slope class 1	Slope class 2	Slope class 3
Low separation and *N* = 300							
0	Nothing constrained	.00	.01	.00	.00	.03	.01
1A	Residual variance time constrained	.00	.01	.00	.00	**.10**	.08
1B	Residual variance classes constrained	.01	.03	.00	.00	**.10**	.00
1C	Random effects constrained	.01	.01	.02	.01	.00	.04
2A	Residual variance constrained time and classes	.01	.04	.00	.00	.22	.03
2B	Random effects and residual variance time constrained	.01	.02	.01	.01	.03	**.20**
2C	Random effects and residual variance classes constrained	**.13**	**.29**	.03	.08	**1.23**	**.37**
Low separation and N = 100							
0	Nothing constrained	.01	.05	.01	.01	.04	**.18**
1A	Residual variance time constrained	.01	.02	.01	.01	.08	0
1B	Residual variance classes constrained	.01	.05	.01	0	.07	**.18**
1C	Random effects constrained	.02	.02	.02	.01	.06	.07
2A	Residual variance constrained time and classes	.02	.07	.01	.02	**.37**	.02
2B	Random effects and residual variance time constrained	.01	.03	.01	.01	.08	**.26**
2C	Random effects and residual variance classes constrained	**.13**	**.19**	.01	.03	**1.06**	**.47**

Relative bias: bias divided by true population value

Codes for relative bias: Bold ≥ .1

The variance ratio in Scenario 1 is 1:3 across classes and over time points, which means that the variance in the first class/time-point is three times smaller compared to the last class/time-point

#### Sample size of 100 and small separation between classes

For a sample size of 100 and a small separation between classes, no model consistently performed best (Fig. 2d, Table 3). Class recovery was highest for the model with constrained random effects across classes and residual variance over time (2B), while classification accuracy was highest for the unconstrained model. Regarding fit, findings were more homogenous, as the BIC, aBIC and AIC indicated that either the unconstrained model or the model with random effects constrained (1C) fitted the data best. The “classes-constrained” model performed poorly, with class recovery ranging from 6.1–21.4% (Supplementary Table 2, Fig. 2d).

### Verification of simulation results with empirical data: the TRacking adolescents’ individual lives survey

#### Main analyses

Of all fitted models, the 5-class solution of the model which constrained the residual variances over time (Model 1A) showed the lowest BIC, AIC and aBIC (Table 4, Supplementary Table 4), followed by the 3-class solution of the unconstrained model. The 4 and 5-class solutions did not converge for the unconstrained model, the model with constrained residual variances across classes (1B) and the model with constrained random effects (1C). Overall, the fit indices indicated that the 3-class models provide a better fit than the 2-class models. Since not all 4-class and 5-class models converged, we focused on the 3-class solutions, to be able to compare all models. The LMR-LRT and VLMR-LRT were significant (*p* < .05) for almost all models and number of classes (except for the 3-class solution for model 1B and the 4-class solution for model 2A).

**Table 4:** Model fit Indices for growth mixture models of aggressive behavior in the TRacking Adolescent Individuals’ Lives Survey (TRAILS), The Netherlands, 2001–2017: text bolded for the best fitting model for each number of classes

Model	# Classes	BIC	aBIC	AIC	Entropy
0: Unconstrained	1^a^	**− 2326**	**− 2367**	**− 2400**	–
	2	**− 4393**	**− 4475**	**− 4541**	0.69
	3	**− 4885**	**− 5009**	**−5107**	0.67
1A: Residual variance time constrained	1^b^	− 2300	− 2325	− 2345	–
	2	− 4090	− 4141	− 4182	0.7
	3	− 4553	− 4630	− 4690	0.65
	4	**− 4738**	**− 4840**	**− 4921**	0.66
	5	**− 4929**	**− 5056**	**− 5157**	0.68
1B: Residual variance classes constrained	2	− 3421	− 3506	− 3575	0.56
	3	− 3149	− 3235	− 3303	0.54
	4	− 3261	− 3369	− 3455	0.54
1C: Random effects constrained	2	− 4143	− 4219	− 4280	0.67
	3	− 4791	− 4902	− 4991	0.66
2A: Residual variance constrained time and classes	2	− 2873	− 2921	− 2959	0.46
	3	− 3029	− 3099	− 3154	0.58
	4	− 3186	− 3278	− 3352	0.55
	5	− 3360	− 3474	− 3565	0.59
2B: Random effects and residual variance time constrained	2	− 3843	− 3888	− 3923	0.66
	3	− 4459	− 4522	− 4573	0.7
	4	− 4685	− 4768	− 4833	0.71
	5	− 4889	−4991	− 5072	0.71
2C: Random effects and residual variance classes constrained	2	− 2705	− 2763	− 2808	**0.79**
	3	− 2916	− 2989	− 3047	**0.78**
	4	− 3032	− 3121	− 3192	**0.76**
	5	− 3155	− 3260	− 3344	**0.73**

For some models, solutions are not shown for all number of classes because in those instances no solutions could be computed: In Model 0 and 1C for 4 and 5 classes, and in Model 1B for 5 classes

*BIC* Bayesian Information Criterion, *aBIC* Adjusted BIC, *AIC* Aikake Information Criterion

^a^This model is the same as the 1-class models 1B, 1C and 2C,

^b^This model is the same as the 1-class models 2A and 2B

**Fig. 3:**
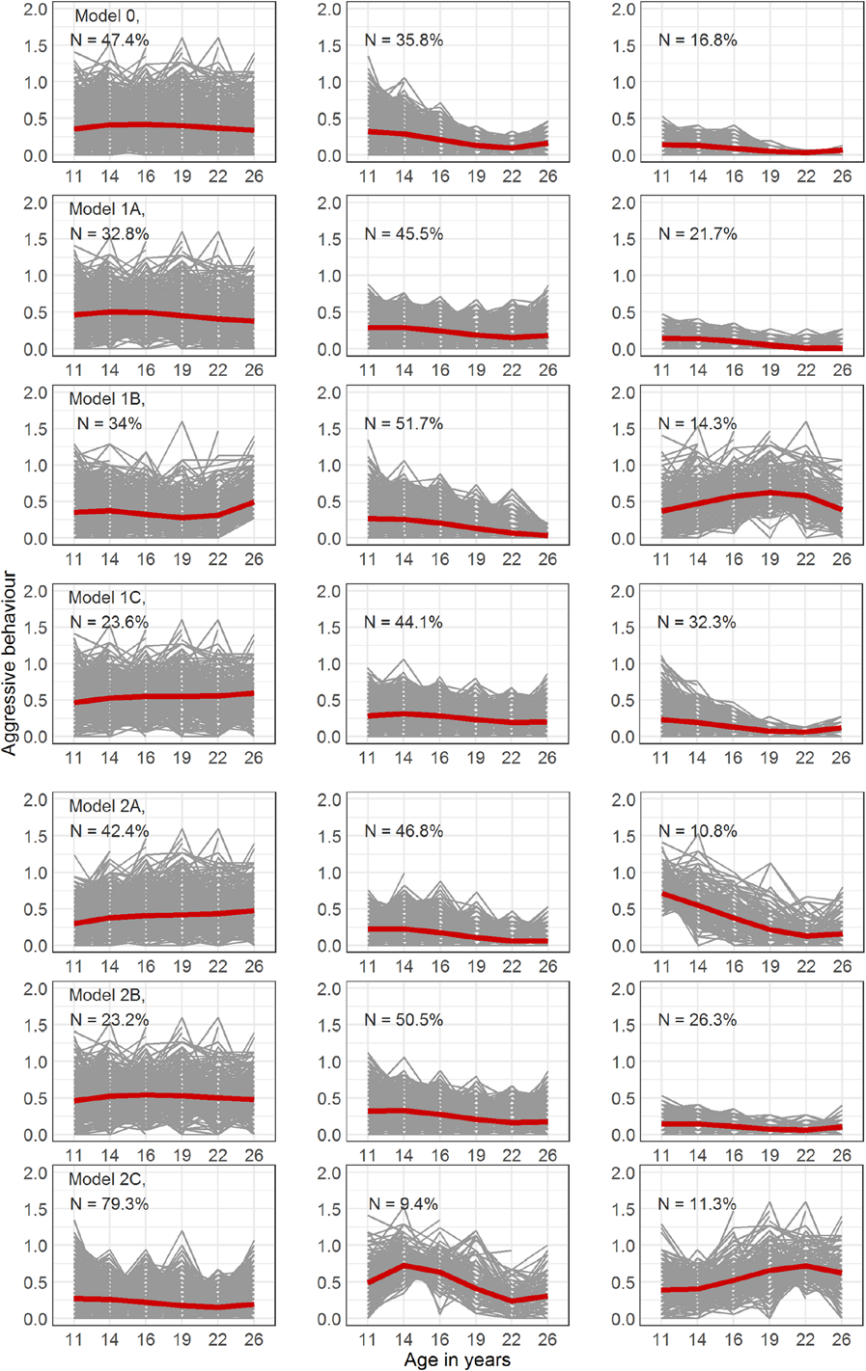
Estimated (red/thick line) and observed (thin/grey lines) trajectories for restricted and freely estimated models of Aggressive Behavior in TRacking Adolescent Individuals’ Lives Survey (TRAILS). The percentages represent the relative class sizes per model

The unconstrained 3-class solution consisted of a high variety stable class (47.4%), a class with decreasing means over time (35.8%) and a low decreasing class (16.8%) (Fig. 3, Table 5). The optimal solution for the model with constrained residual variances over time (1A), constrained random effects (1C) and constrained random effects and residual variances over time (2B) showed a similar structure to the unconstrained model, with a stable class with little variation instead of the decreasing class. The optimal solution for the model with constrained residual variance across classes (1B) showed a similar increasing and decreasing class, but the last class was different: increasing with a peak around age 19. The optimal solution for the model with residual variance constrained over time and across classes (2A) also had a different last class: characterized by a high intercept and strongly decreasing over time. The optimal solution for the “classes-constrained model” showed a substantially different class structure, with one class peaking around age 14 (9.4%) and another around age 22 (11.3%) and a third class being stable over time (79.3%).

**Table 5:** Parameter Estimates of the 3-Class Solution in the TRacking Adolescent Individuals’ Lives Survey (TRAILS), The Netherlands, 2001–2017

Model	Parameter	Class 1		Class 2		Class 3	
		Mean	Variance	Mean	Variance	Mean	Variance
0: Unconstrained	Intercept	0.358	0.024	0.325	0.008	0.145	0.009
	Linear Slope	0.087	0.001	0.008	0.001	0.005	0.001
	Quadratic Slope	−0.033	0^a^	−0.048	0^a^	−0.024	0^a^
	Cubic slope	0.003	0^a^	0.008	0^a^	0.004	0^a^
	T1	–	0.038	–	0.049	–	0.01
	T2	–	0.04	–	0.03	–	0.008
	T3	–	0.041	–	0.019	–	0.01
	T4	–	0.059	–	0.01	–	0.002
	T5	–	0.05	–	0.006	–	0.001
	T6	–	0.049	–	0.011	–	0.001
1A: Residual variance time constrained	Intercept	0.460	0.012	0.283	0.008	0.136	0.011
	Linear Slope	0.078	0.003	0.039	0.001	0.024	0.001
	Quadratic Slope	−0.039	0^a^	− 0.042	0^a^	− 0.03	0^a^
	Cubic slope	0.004	0^a^	0.006	0^a^	0.004	0^a^
	T1	–	0.058	–	0.023	–	0.005
	T2	–	0.058	–	0.023	–	0.005
	T3	–	0.058	–	0.023	–	0.005
	T4	–	0.058	–	0.023	–	0.005
	T5	–	0.058	–	0.023	–	0.005
	T6	–	0.058	–	0.023	–	0.005
1B: Residual variance classes constrained	Intercept	0.352	0.026	0.265	0.022	0.369	0.037
	Linear Slope	0.078	0.003	0.019	0.001	0.089	0.003
	Quadratic Slope	−0.07	0^a^	−0.033	0^a^	0.023	0^a^
	Cubic slope	0.012	0^a^	0.004	0^a^	− 0.008	0^a^
	T1	–	0.034	–	0.034	–	0.034
	T2	–	0.029	–	0.029	–	0.029
	T3	–	0.028	–	0.028	–	0.028
	T4	–	0.027	–	0.027	–	0.027
	T5	–	0.026	–	0.026	–	0.026
	T6	–	0.005	–	0.005	–	0.005
1C: Random effects constrained	Intercept	0.282	0.013	0.466	0.013	0.234	0.013
	Linear Slope	0.073	0.001	0.091	0.001	−0.023	0.001
	Quadratic Slope	−0.048	0^a^	−0.033	0^a^	− 0.025	0^a^
	Cubic slope	0.006	0^a^	0.004	0^a^	0.005	0*
	T1	–	0.027	–	0.067	–	0.031
	T2	–	0.029	–	0.061	–	0.013
	T3	–	0.027	–	0.057	–	0.011
	T4	–	0.028	–	0.094	–	0.003
	T5	–	0.023	–	0.09	–	0.001
	T6	–	0.03	–	0.075	–	0.004
Model 2A: Residual variance Constrained time and classes	Intercept	0.302	0.021	0.223	0.006	0.715	0.021
	Linear Slope	0.105	0.001	0.043	0.000^b^	−0.142	0.000^b^
	Quadratic Slope	−0.034	0^a^	−0.045	0^a^	−0.029	0^a^
	Cubic slope	0.004	0^a^	0.006	0^a^	0.007	0^a^
	T1	–	0.028	–	0.028	–	0.028
	T2	–	0.028	–	0.028	–	0.028
	T3	–	0.028	–	0.028	–	0.028
	T4	–	0.028	–	0.028	–	0.028
	T5	–	0.028	–	0.028	–	0.028
	T6	–	0.028	–	0.028	–	0.028
2B: Random effects and residual variance time constrained	Intercept	0.463	0.01	0.323	0.01	0.148	0.01
	Linear Slope	0.094	0.001	0.04	0.001	0.026	0.001
	Quadratic Slope	−0.033	0^a^	−0.044	0^a^	−0.032	0^a^
	Cubic slope	0.003	0^a^	0.006	0^a^	0.005	0^a^
	T1	–	0.073	–	0.029	–	0.006
	T2	–	0.073	–	0.029	–	0.006
	T3	–	0.073	–	0.029	–	0.006
	T4	–	0.073	–	0.029	–	0.006
	T5	–	0.073	–	0.029	–	0.006
	T6	–	0.073	–	0.029	–	0.006
2C: Random effects and residual variance classes constrained	Intercept	0.273	0.018	0.491	0.018	0.397	0.018
	Linear Slope	0.019	0.001	0.469	0.001	−0.074	0.001
	Quadratic Slope	−0.032	0^a^	−0.261	0^a^	0.099	0^a^
	Cubic slope	0.005	0^a^	0.032	0^a^	−0.015	0^a^
	T1	–	0.037	–	0.037	–	0.037
	T2	–	0.023	–	0.023	–	0.023
	T3	–	0.026	–	0.026	–	0.026
	T4	–	0.033	–	0.033	–	0.033
	T5	–	0.017	–	0.017	–	0.017
	T6	–	0.027	–	0.027	–	0.027

^a^These variances were manually set to be equal to zero

^b^These variances were estimated to be very close to zero, which led to a warning that the covariance matrix was not positive definite. Nevertheless, the variances were not set to be equal to zero, in order to keep the model comparable to other models

#### Sensitivity analyses

Overall, the 2-class and 3-class results of the 3 subsamples of 300 individuals were similar to the results for the whole dataset, while the class sizes differed from those in the whole dataset. For all 3-class models, except the model with residual variance constrained over time and classes (2A) and the “classes-constrained model”, the same class structure was found. Among the 2-class models, only Model 2A showed a different class structure. Like in the main analyses, the BIC, AIC and aBIC indicated that the unconstrained 3-class model fitted the data best.

## Discussion

This simulation study showed that the unconstrained model performed best in terms of class recovery, bias and class assignment, if the variation across classes and over time differs and the sample size is sufficient (*N* = 1000). In all scenarios and for all sample sizes, the model with both random variances and residual variances constrained to be equal across classes (“classes-constrained model”) performed the poorest. For smaller sample sizes (100 or 300), no model consistently performed best. Analyses of the TRAILS cohort confirmed that the “classes-constrained model” tended to result in classes that substantially differed from the classes resulting from the other models.

This study showed that constraining variance parameters to be equal, when they are not, induces bias and reduces class recovery and classification accuracy, especially for the “classes-constrained model”. These findings are in accordance with recent studies which focused on residual variances [5, 14, 15] or random effects [30]. More specifically, we confirmed the finding by Davies and Glonek that the unconstrained model performs best and the classes constrained model performs the poorest [10]. Contrary to our findings, some authors found no [9] or a positive effect [31] of residual variance constraints on model performance. This difference might be due to different evaluation criteria: while we focused on class recovery, bias and class assignment, these studies focused on the BIC indicating the correct number of classes. Finding the correct number of classes might not always be affected by model misspecification, but parameter estimates usually are [32]. Selecting the correct number of classes is a suboptimal evaluation criterion, since a model with correct number of classes can still be a poor representation of the simulated classes, for instance when almost empty classes are estimated.

For all sample sizes and in all scenarios, the “classes-constrained” model performed poorly. This may be explained by the fact that this is the only model that constrained both types of variances across classes, resulting in a model which assumes the overall variance to be equal across classes. Therefore, spurious classes, which are equal in terms of variance, might be found when variances differ across classes. Classes can be based not just on the trajectories themselves, but also on the variation around the trajectory [1]. For instance in school performance, a class of high achievers might show less variation than a class of lower achieving students which differ more between measurements [17].

Model performance by variance misspecification was substantially influenced by sample size, with a sample size of 1000 leading to good performance and sample size of 300 or less leading to relatively poor performance. In most scenarios, the unconstrained model still performed best for a sample size of 300. For a sample size of 100, there was no model that performed consistently best. Negative variances and low class recovery were omnipresent for sample sizes of 100 and 300. Our results do not indicate that a sample size of 1000 is always sufficient. We aimed to mimic empirical data as closely as possible, by basing our population values on an earlier review of empirical studies applying GMM. However, we based our variance ratios on findings in 2 empirical studies, since variance estimates are rarely reported in GMM.

To the best of our knowledge this is the first study that focused on all possible variance parameter constraints within GMM. The findings may help researchers decide which variance constrains should be avoided. We combined simulated and empirical data and used a literature review to specify the simulated data to make it resemble empirical data. Another possible limitation is the focus in the simulation study on linear models only, which are relatively simple. Therefore, the results of our simulation study might be more positive than in empirical data. Future work could focus on variance specification of more complex GMMs, such as cubic growth GMMs and second order GMMs [33]. This research paper focused on GMM, but our results also apply to Latent Class Growth Analysis (LCGA) [34]. LCGA could be considered to be a special case of GMM, without random effects. Residual variance specification across time and classes will potentially modify the estimations of LCGA.

**Fig. 4:**
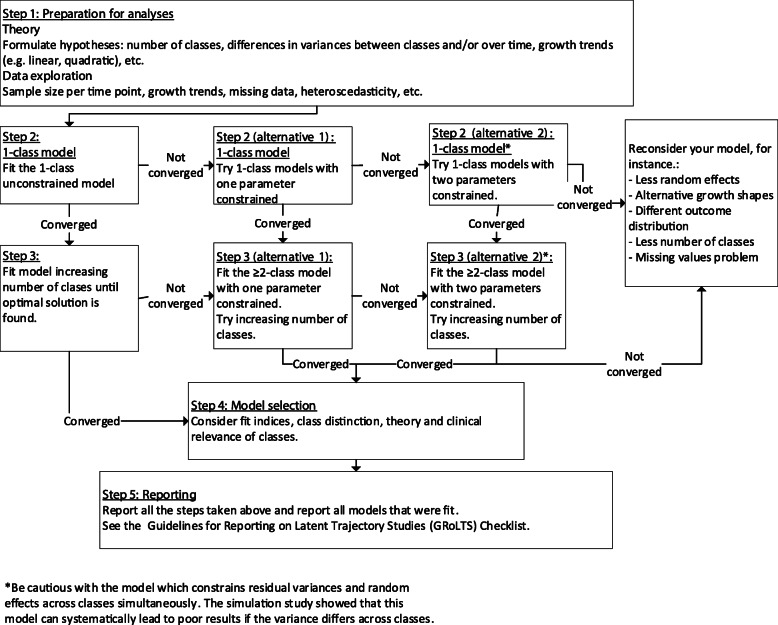
Flowchart to aid the decision making process of which variance parameters could be constrained in Growth Mixture Modeling

Based on the current study, we provide four recommendations for analyzing GMMs in general and for constraining variance parameters in GMM, which are also summarized in the flow-chart as depicted in Fig. 4. First, our study confirmed that if residual variances differ over time and classes and random effect variances over classes, the unconstrained model is usually preferred. Therefore, in case of adequate sample sizes, we recommend to start with the unconstrained model. Second, the model which constrains both the random effects and residual variances across classes (the classes constrained model) rarely recovered the simulated classes and led to substantially different results for the TRAILS sample. Therefore, we discourage the use of this model. It should be noted that in the Mplus software package [20], the default settings correspond to this model. The researcher should determine which model(s) should be estimated, and should not rely on default values in software packages. Defaults are only created for writing syntax parsimoniously, and are not to be considered the most suitable statistical model specifications. Third, we advise to always try different variance specifications, especially if the unconstrained model does not converge without issues. Knowledge of the theory and data may suggest which variance restrictions should be applied. Selection of the final model and the optimal number of classes should be based on fit indices (e.g. BIC, aBIC), separation between classes, the LMR-LRT and bootstrapped LRT, and the interpretation (sensibility and distinctiveness) of the classes. Fourth, it is of utmost importance to report which variances were constrained or let free to aid transparency and replicability. The fact that variances structures are hardly reported makes us suspect that the default (most-restricted) variance specification has been used, while this model consistently shows the poorest results in terms of fit and bias. Inadequate or incomplete reporting of the results for latent trajectory analysis hampers interpretation and critical appraisal of results, as well as comparison of results between studies [7]. We reemphasize the importance of following the GRoLTS-Checklist when conducting and reporting GMM analyses, especially regarding variance specifications.

## Conclusions

We conclude that it is best to not constrain any variance parameters in GMM. But if convergence issues arise, one might constrain one variance parameter (see Fig. 4 for a flowchart to aid the decision process). No generalizable conclusions can be drawn about which are the best variance parameters to be constrained when the unconstrained model does not converge properly. Theories, previous conceptual knowledge and fit information should guide the decision process. Researchers are encouraged to try different variance specifications and to report these all clearly. However, constraining the residual variances and the random effect variances to be equal across classes simultaneously, could seriously bias the results, when they are different. Therefore, constraining both random effect variances and residual variances across classes is discouraged.

## Supplementary Information

**Additional file 1: Table S1.** How often was each model selected to be best fitting according to several fit indices in the simulation study: text bolded for the lowest (information criteria) or highest (entropy) value. **Table S2.** Class recovery, occurrence of negative variances and individuals correctly classified over 1000 replications, by sample sizes (N), degrees of separation, and ratios of residual variances (var.) and random effects in the data generation process. **Table S3A.** Absolute value of relative bias of intercept (int) and slope per simulated scenario and analysis model: findings over the 1000 replications in the simulation for a sample size of 1000 and a high degree of separation between classes. **Table S3B.** Absolute value of relative bias of intercept (int) and slope per simulated scenario and analysis model: findings over the 1000 replications in the simulation for a sample size of 1000 and a low degree of separation between classes. **Table S3C.** Absolute value of relative bias of intercept (int) and slope per simulated scenario and analysis model: findings over the 1000 replications in the simulation for a sample size of 300 and a low degree of separation between classes. **Table S3D.** Absolute value of relative bias of intercept (int) and slope per simulated scenario and analysis model: findings over the 1000 replications in the simulation for a sample size of 100 and a low degree of separation between classes. **Table S4.** Model fit Indices for growth mixture models of aggressive behavior in the TRacking Adolescent Individuals’ Lives Survey (TRAILS), The Netherlands, 2001–2017, by model. **Figure S1.** Line plots for the first of the 1000 datasets in the simulated data in scenario 1.

## Data Availability

The TRIALS dataset analysed during the current study are available and can be requested at www.trails.nl. The R code to simulate and analyze the data can be found at https://osf.io/68ptf/?view_only=4e3bd94405b3427498449208d02a7b88

## Abbreviations

aBIC: Sample-size Adjusted BIC
AIC: Akaike Information Criterion
ASR: Adult Self-report
BIC: Bayesian Information Criterion
GMM: Growth Mixture Modeling
TRAILS: TRacking Adolescent Individuals’ Lives Survey
YSR: Youth Self-report
