# Supplementary material for: Variance constraints strongly influenced model performance in growth mixture modeling: a simulation and empirical study
Source: BMC Med Res Methodol. 2020 Nov 12;20:276. doi: 10.1186/s12874-020-01154-0 (PMC7659099; doi:10.1186/s12874-020-01154-0)

| Supplementary Table 1 How often was each model selected to be best fitting according to several fit indices in the simulation study: text bolded for the lowest (information criteria) or highest (entropy) value. | | | | | | | | | | | | | | | | | | |  |
| --- | --- | --- | --- | --- | --- | --- | --- | --- | --- | --- | --- | --- | --- | --- | --- | --- | --- | --- | --- |
|  |  | N = 1000, large separation | | | | |  |  |  |  | N = 1000, small separation | | | | |  |  |  | |
|  |  | Model | | |  |  |  |  |  |  | Model | | |  |  |  |  |  | |
| Scenario |  | 0 | 1A | 1B | 1C | 2A | 2B | 2C | Missings^a^ |  | 0 | 1A | 1B | 1C | 2A | 2B | 2C | Missings^a^ ^aa^a | |
| 1 | BIC | **1.00** | .00 | .00 | .00 | .00 | .00 | .00 | .00 |  | **1.00** | .00 | .00 | .00 | .00 | .00 | .00 | .00 | |
|  | aBIC | **1.00** | .00 | .00 | .00 | .00 | .00 | .00 | .00 |  | **1.00** | .00 | .00 | .00 | .00 | .00 | .00 | .00 | |
|  | AIC | **1.00** | .00 | .00 | .00 | .00 | .00 | .00 | .00 |  | **1.00** | .00 | .00 | .00 | .00 | .00 | .00 | .00 | |
|  | Entropy | .00 | .00 | .00 | .00 | .25 | .00 | **.75** | .00 |  | .00 | .02 | .18 | .00 | **.81** | .00 | .00 | .00 | |
| 2 | BIC | **1.00** | .00 | .00 | .00 | .00 | .00 | .00 | .00 |  | **1.00** | .00 | .00 | .00 | .00 | .00 | .00 | .00 | |
|  | aBIC | **1.00** | .00 | .00 | .00 | .00 | .00 | .00 | .00 |  | **1.00** | .00 | .00 | .00 | .00 | .00 | .00 | .00 | |
|  | AIC | **1.00** | .00 | .00 | .00 | .00 | .00 | .00 | .00 |  | **1.00** | .00 | .00 | .00 | .00 | .00 | .00 | .00 | |
|  | Entropy | .00 | .37 | .00 | .00 | .05 | .00 | **.58** | .00 |  | .00 | .15 | .01 | .00 | **.83** | .00 | .00 | .00 | |
| 3 | BIC | **1.00** | .00 | .00 | .00 | .00 | .00 | .00 | .00 |  | **1.00** | .00 | .00 | .00 | .00 | .00 | .00 | .00 | |
|  | aBIC | **1.00** | .00 | .00 | .00 | .00 | .00 | .00 | .00 |  | **1.00** | .00 | .00 | .00 | .00 | .00 | .00 | .00 | |
|  | AIC | **1.00** | .00 | .00 | .00 | .00 | .00 | .00 | .00 |  | **1.00** | .00 | .00 | .00 | .00 | .00 | .00 | .00 | |
|  | Entropy | .00 | .07 | .01 | .00 | .42 | .00 | **.51** | .00 |  | .00 | .00 | .05 | .00 | **.95** | .00 | .00 | .00 | |
| 4 | BIC | **1.00** | .00 | .00 | .00 | .00 | .00 | .00 | .00 |  | **1.00** | .00 | .00 | .00 | .00 | .00 | .00 | .00 | |
|  | aBIC | **1.00** | .00 | .00 | .00 | .00 | .00 | .00 | .00 |  | **1.00** | .00 | .00 | .00 | .00 | .00 | .00 | .00 | |
|  | AIC | **1.00** | .00 | .00 | .00 | .00 | .00 | .00 | .00 |  | **1.00** | .00 | .00 | .00 | .00 | .00 | .00 | .00 | |
|  | Entropy | .00 | .86 | .00 | .00 | .02 | .00 | .12 | .00 |  | .00 | .01 | .00 | .00 | **.99** | .00 | .00 | .00 | |
| 5 | BIC | **1.00** | .00 | .00 | .00 | .00 | .00 | .00 | .00 |  | **1.00** | .00 | .00 | .00 | .00 | .00 | .00 | .00 | |
|  | aBIC | **1.00** | .00 | .00 | .00 | .00 | .00 | .00 | .00 |  | **1.00** | .00 | .00 | .00 | .00 | .00 | .00 | .00 | |
|  | AIC | **1.00** | .00 | .00 | .00 | .00 | .00 | .00 | .00 |  | **1.00** | .00 | .00 | .00 | .00 | .00 | .00 | .00 | |
|  | Entropy | .00 | .00 | .03 | .00 | .37 | .00 | **.59** | .00 |  | .00 | .00 | .21 | .00 | **.79** | .00 | .00 | .00 | |
| 6 | BIC | **1.00** | .00 | .00 | .00 | .00 | .00 | .00 | .00 |  | **1.00** | .00 | .00 | .00 | .00 | .00 | .00 | .00 | |
|  | aBIC | **1.00** | .00 | .00 | .00 | .00 | .00 | .00 | .00 |  | **1.00** | .00 | .00 | .00 | .00 | .00 | .00 | .00 | |
|  | AIC | **1.00** | .00 | .00 | .00 | .00 | .00 | .00 | .00 |  | **1.00** | .00 | .00 | .00 | .00 | .00 | .00 | .00 | |
|  | Entropy | .00 | .00 | .07 | .00 | .54 | .00 | .40 | .00 |  | .00 | .00 | .13 | .00 | **.87** | .00 | .00 | .00 | |
| 7 | BIC | **1.00** | .00 | .00 | .00 | .00 | .00 | .00 | .00 |  | **1.00** | .00 | .00 | .00 | .00 | .00 | .00 | .00 | |
|  | aBIC | **1.00** | .00 | .00 | .00 | .00 | .00 | .00 | .00 |  | **1.00** | .00 | .00 | .00 | .00 | .00 | .00 | .00 | |
|  | AIC | **1.00** | .00 | .00 | .00 | .00 | .00 | .00 | .00 |  | **1.00** | .00 | .00 | .00 | .00 | .00 | .00 | .00 | |
|  | Entropy | .00 | .00 | .00 | .00 | .20 | .00 | **.80** | .00 |  | .00 | .00 | .02 | .00 | **.98** | .00 | .00 | .00 | |
| 8 | BIC | **1.00** | .00 | .00 | .00 | .00 | .00 | .00 | .00 |  | **1.00** | .00 | .00 | .00 | .00 | .00 | .00 | .00 | |
|  | aBIC | **1.00** | .00 | .00 | .00 | .00 | .00 | .00 | .00 |  | **1.00** | .00 | .00 | .00 | .00 | .00 | .00 | .00 | |
|  | AIC | **1.00** | .00 | .00 | .00 | .00 | .00 | .00 | .00 |  | **1.00** | .00 | .00 | .00 | .00 | .00 | .00 | .00 | |
|  | Entropy | .00 | .01 | .00 | .00 | **.60** | .00 | .39 | .00 |  | .00 | .00 | .00 | .00 | **1.00** | .00 | .00 | .00 | |
| a) ‘Missings’ indicates how often the fit index could not be calculated for at least one model, and therefore that particular model could not have been selected as the best fitting model.  b) Model 0: Nothing constrained, Model 1A: Residual variance time constrained, Model 1B: Residual variance classes constrained, Model 1C: Random effects constrained, Model 2A: Residual variance constrained time and classes, Model 2B: Random effects and residual variance time constrained, Model 2C: Random effects and residual variance classes constrained (Mplus default). | | | | | | | | | | | | | | | | | | | |

| Supplementary Table 2. Class recovery, occurrence of negative variances and individuals correctly classified over 1000 replications, by sample sizes (N), degrees of separation, and ratios of residual variances (var.) and random effects in the data generation process. | | | | | | | | | | | | | | | | | | | | | | | | | | |  |
| --- | --- | --- | --- | --- | --- | --- | --- | --- | --- | --- | --- | --- | --- | --- | --- | --- | --- | --- | --- | --- | --- | --- | --- | --- | --- | --- | --- |
|  | Ratios of variances in data generation | | | | Number of valid replications, number of valid replications with negative variances, % of individuals which are classified in their correct class | | | | | | | | | | | | | | | | | | | | | |  |
| N, separation | | Residual  var. classes | Residual var. time | Random effects | | Model 0:  Nothing constrained | | | Model 1A:  Residual variance time constrained | | | Model 1B:  Residual variance classes constrained | | | Model 1C:  Random effects constrained | | | Model 2A:  Residual variance constrained time and classes | | | Model 2B:  Random effects and residual variance time constrained | | | Model 2C:  Random effects and residual variance classes constrained  (Mplus default) | | | |
|  | |  |  |  | | Class reco. | neg. var. | Class accur | Class reco. | neg. var. | Corr.  Class | Class reco. | neg. var. | Class accur | Class reco. | neg. var. | Class accur | Class reco. | neg. var. | Class accur | Class reco. | neg. var. | Corr.  Class | Class reco. | neg. var. | Class accur | |
| 1000, | | 1:3 | 1:3 | 1:3 | | 1.00 | 0.00 | 0.97 | 1.00 | 0.35 | 0.96 | 1.00 | 1.00 | 0.96 | 1.00 | 0.00 | 0.96 | 1.00 | 1.00 | 0.95 | 1.00 | 0.10 | 0.96 | 0.00 | 0.88 | 0.80 | |
| high | | 1:3 | 1:10 | 1:3 | | 1.00 | 0.33 | 0.97 | 1.00 | 1.00 | 0.96 | 1.00 | 1.00 | 0.96 | 1.00 | 0.03 | 0.96 | 1.00 | 1.00 | 0.96 | 1.00 | 1.00 | 0.96 | 0.00 | 0.90 | - | |
|  | | 1:3 | 1:3 | 1:10 | | 1.00 | 0.20 | 0.98 | 1.00 | 1.00 | 0.94 | 1.00 | 1.00 | 0.97 | 1.00 | 0.63 | 0.96 | 1.00 | 1.00 | 0.96 | 1.00 | 1.00 | 0.96 | 0.00 | 0.13 | - | |
|  | | 1:3 | 1:10 | 1:10 | | 1.00 | 0.00 | 0.98 | 1.00 | 0.43 | 0.97 | 1.00 | 1.00 | 0.96 | 1.00 | 0.00 | 0.97 | 1.00 | 1.00 | 0.96 | 1.00 | 0.30 | 0.97 | 0.00 | 1.00 | - | |
|  | | 1:10 | 1:3 | 1:3 | | 1.00 | 0.03 | 0.98 | 1.00 | 0.92 | 0.95 | 1.00 | 1.00 | 0.97 | 1.00 | 0.56 | 0.97 | 1.00 | 1.00 | 0.96 | 1.00 | 0.54 | 0.96 | 0.31 | 0.07 | 0.94 | |
|  | | 1:10 | 1:3 | 1:10 | | 1.00 | 0.01 | 0.98 | 1.00 | 0.95 | 0.98 | 1.00 | 1.00 | 0.96 | 1.00 | 0.00 | 0.98 | 1.00 | 1.00 | 0.96 | 1.00 | 0.00 | 0.97 | 0.00 | 1.00 | - | |
|  | | 1:10 | 1:10 | 1:3 | | 1.00 | 0.10 | 0.99 | 1.00 | 1.00 | 0.97 | 1.00 | 1.00 | 0.97 | 1.00 | 0.27 | 0.98 | 1.00 | 1.00 | 0.96 | 1.00 | 0.00 | 0.96 | 0.00 | 0.97 | 0.93 | |
|  | | 1:10 | 1:10 | 1:10 | | 1.00 | 0.02 | 0.98 | 1.00 | 0.95 | 0.96 | 1.00 | 1.00 | 0.97 | 1.00 | 0.08 | 0.98 | 1.00 | 1.00 | 0.96 | 1.00 | 0.84 | 0.96 | 0.00 | 0.98 | - | |
|  | |  |  |  | |  |  |  |  |  |  |  |  |  |  |  |  |  |  |  |  |  |  |  |  |  | |
| 1000, | | 1:3 | 1:3 | 1:3 | | 1.00 | 0.01 | 0.74 | 1.00 | 0.33 | 0.52 | 1.00 | 1.00 | 0.74 | 1.00 | 0.00 | 0.51 | 1.00 | 1.00 | 0.50 | 1.00 | 0.04 | 0.61 | 0.00 | 0.06 | - | |
| low | | 1:3 | 1:10 | 1:3 | | 1.00 | 0.33 | 0.66 | 1.00 | 1.00 | 0.54 | 1.00 | 1.00 | 0.65 | 1.00 | 0.01 | 0.48 | 1.00 | 1.00 | 0.68 | 1.00 | 1.00 | 0.65 | 0.00 | 0.03 | - | |
|  | | 1:3 | 1:3 | 1:10 | | 1.00 | 0.23 | 0.73 | 1.00 | 1.00 | 0.52 | 1.00 | 1.00 | 0.54 | 1.00 | 0.86 | 0.55 | 1.00 | 1.00 | 0.67 | 1.00 | 1.00 | 0.63 | 0.00 | 0.01 | - | |
|  | | 1:3 | 1:10 | 1:10 | | 1.00 | 0.00 | 0.64 | 1.00 | 0.41 | 0.50 | 1.00 | 1.00 | 0.50 | 1.00 | 0.00 | 0.59 | 1.00 | 1.00 | 0.57 | 1.00 | 0.27 | 0.71 | 0.00 | 0.82 | - | |
|  | | 1:10 | 1:3 | 1:3 | | 1.00 | 0.05 | 0.77 | 1.00 | 0.87 | 0.49 | 1.00 | 1.00 | 0.74 | 1.00 | 0.75 | 0.63 | 1.00 | 1.00 | 0.52 | 1.00 | 0.06 | 0.64 | 0.00 | 0.00 | - | |
|  | | 1:10 | 1:3 | 1:10 | | 1.00 | 0.01 | 0.67 | 1.00 | 0.96 | 0.52 | 1.00 | 1.00 | 0.70 | 1.00 | 0.00 | 0.69 | 1.00 | 1.00 | 0.64 | 1.00 | 0.00 | 0.66 | 0.00 | 0.96 | - | |
|  | | 1:10 | 1:10 | 1:3 | | 1.00 | 0.13 | 0.76 | 1.00 | 1.00 | 0.52 | 1.00 | 1.00 | 0.66 | 1.00 | 0.30 | 0.81 | 1.00 | 1.00 | 0.57 | 1.00 | 0.00 | 0.70 | 0.00 | 0.66 | - | |
|  | | 1:10 | 1:10 | 1:10 | | 1.00 | 0.03 | 0.75 | 1.00 | 0.98 | 0.44 | 1.00 | 1.00 | 0.60 | 1.00 | 0.08 | 0.71 | 1.00 | 1.00 | 0.52 | 1.00 | 0.74 | 0.74 | 0.00 | 0.53 | - | |
|  | |  |  |  | |  |  |  |  |  |  |  |  |  |  |  |  |  |  |  |  |  |  |  |  |  | |
| 300, | | 1:3 | 1:3 | 1:3 | | 0.89 | 0.33 | 0.78 | 0.98 | 0.74 | 0.53 | 0.86 | 1.00 | 0.56 | 1.00 | 0.03 | 0.51 | 0.81 | 1.00 | 0.47 | 1.00 | 0.21 | 0.61 | 0.01 | 0.23 | 0.52 | |
| Low | | 1:3 | 1:10 | 1:3 | | 0.98 | 0.57 | 0.71 | 1.00 | 1.00 | 0.56 | 0.98 | 1.00 | 0.47 | 1.00 | 0.15 | 0.50 | 0.96 | 1.00 | 0.53 | 1.00 | 0.95 | 0.60 | 0.00 | 0.18 | - | |
|  | | 1:3 | 1:3 | 1:10 | | 1.00 | 0.63 | 0.74 | 0.99 | 1.00 | 0.52 | 1.00 | 1.00 | 0.53 | 0.96 | 0.71 | 0.53 | 0.97 | 1.00 | 0.55 | 1.00 | 1.00 | 0.61 | 0.00 | 0.12 | 0.63 | |
|  | | 1:3 | 1:10 | 1:10 | | 1.00 | 0.14 | 0.68 | 1.00 | 0.68 | 0.54 | 1.00 | 1.00 | 0.47 | 1.00 | 0.03 | 0.65 | 1.00 | 1.00 | 0.57 | 1.00 | 0.42 | 0.68 | 0.00 | 0.78 | 0.54 | |
|  | | 1:10 | 1:3 | 1:3 | | 0.98 | 0.47 | 0.80 | 0.90 | 0.92 | 0.48 | 0.94 | 1.00 | 0.57 | 1.00 | 0.66 | 0.58 | 0.76 | 1.00 | 0.46 | 0.99 | 0.22 | 0.58 | 0.01 | 0.07 | 0.53 | |
|  | | 1:10 | 1:3 | 1:10 | | 1.00 | 0.24 | 0.69 | 1.00 | 0.92 | 0.48 | 0.96 | 1.00 | 0.50 | 1.00 | 0.03 | 0.75 | 0.97 | 1.00 | 0.52 | 1.00 | 0.00 | 0.65 | 0.00 | 0.88 | 0.61 | |
|  | | 1:10 | 1:10 | 1:3 | | 1.00 | 0.54 | 0.74 | 1.00 | 1.00 | 0.53 | 0.98 | 1.00 | 0.58 | 1.00 | 0.51 | 0.80 | 0.98 | 1.00 | 0.55 | 1.00 | 0.00 | 0.70 | 0.00 | 0.65 | 0.62 | |
|  | | 1:10 | 1:10 | 1:10 | | 1.00 | 0.36 | 0.74 | 1.00 | 0.95 | 0.50 | 1.00 | 1.00 | 0.61 | 1.00 | 0.26 | 0.72 | 1.00 | 1.00 | 0.57 | 1.00 | 0.66 | 0.68 | 0.00 | 0.59 | - | |
|  | |  |  |  | |  |  |  |  |  |  |  |  |  |  |  |  |  |  |  |  |  |  |  |  |  | |
| 100, | | 1:3 | 1:3 | 1:3 | | 0.39 | 0.93 | 0.72 | 0.61 | 0.95 | 0.54 | 0.56 | 1.00 | 0.55 | 0.84 | 0.43 | 0.52 | 0.43 | 1.00 | 0.47 | 0.98 | 0.43 | 0.61 | 0.10 | 0.48 | 0.53 | |
| low | | 1:3 | 1:10 | 1:3 | | 0.54 | 0.92 | 0.71 | 0.76 | 0.99 | 0.54 | 0.74 | 1.00 | 0.49 | 0.89 | 0.47 | 0.51 | 0.64 | 1.00 | 0.53 | 0.97 | 0.83 | 0.60 | 0.05 | 0.40 | 0.55 | |
|  | | 1:3 | 1:3 | 1:10 | | 0.66 | 0.93 | 0.74 | 0.77 | 1.00 | 0.48 | 0.89 | 1.00 | 0.56 | 0.78 | 0.85 | 0.51 | 0.65 | 1.00 | 0.53 | 0.97 | 0.94 | 0.63 | 0.04 | 0.44 | 0.55 | |
|  | | 1:3 | 1:10 | 1:10 | | 0.87 | 0.73 | 0.74 | 1.00 | 0.90 | 0.51 | 0.87 | 1.00 | 0.51 | 1.00 | 0.25 | 0.70 | 0.82 | 1.00 | 0.56 | 1.00 | 0.51 | 0.63 | 0.04 | 0.81 | 0.57 | |
|  | | 1:10 | 1:3 | 1:3 | | 0.46 | 0.97 | 0.72 | 0.55 | 0.97 | 0.48 | 0.64 | 1.00 | 0.59 | 0.79 | 0.85 | 0.55 | 0.46 | 1.00 | 0.49 | 0.93 | 0.40 | 0.60 | 0.14 | 0.47 | 0.59 | |
|  | | 1:10 | 1:3 | 1:10 | | 0.82 | 0.75 | 0.72 | 0.99 | 0.91 | 0.51 | 0.71 | 1.00 | 0.52 | 1.00 | 0.15 | 0.79 | 0.66 | 1.00 | 0.53 | 1.00 | 0.01 | 0.63 | 0.09 | 0.85 | 0.51 | |
|  | | 1:10 | 1:10 | 1:3 | | 0.81 | 0.93 | 0.74 | 0.99 | 0.99 | 0.52 | 0.70 | 1.00 | 0.60 | 1.00 | 0.75 | 0.80 | 0.66 | 1.00 | 0.54 | 1.00 | 0.01 | 0.62 | 0.12 | 0.82 | 0.57 | |
|  | | 1:10 | 1:10 | 1:10 | | 0.89 | 0.90 | 0.74 | 0.99 | 0.96 | 0.52 | 0.89 | 1.00 | 0.60 | 1.00 | 0.64 | 0.74 | 0.83 | 1.00 | 0.56 | 1.00 | 0.67 | 0.66 | 0.04 | 0.77 | 0.56 | |
| Class accur, classification accuracy; Class reco, Class recovery (proportion of replications in which the simulated classes were recovered); neg. var., negative variances (proportion of replications with at least one negative variance); Residual var., Residual variance  Color codes for class recovery and correct classification: green, ≥.80; yellow, ≥.60 and < .80; red, <.60  1:3 means that the variance in the first class or first time point is three times smaller than in the last class or last time point. 1:10 means that this ratio is 1:10. | | | | | | | | | | | | | | | | | | | | | | | | | | | |

| Supplementary Table3A. Absolute value of relative bias of intercept (int) and slope per simulated scenario and analysis model: findings over the 1000 replications in the simulation for a sample size of 1000 and a high degree of separation between classes. | | | | | | | | | | | | | | | | | |
| --- | --- | --- | --- | --- | --- | --- | --- | --- | --- | --- | --- | --- | --- | --- | --- | --- | --- |
| Residual variance across classes | Residual variance over time | Random effect variances across classes |  | Model 0:  Nothing constrained | | Model 1A:  Residual variance time constrained | | Model 1B:  Residual variance classes constrained | | Model 1C:  Random effects constrained | | Model 2A:  Residual variance time and classes constrained | | Model 2B:  Random effects and residual variance time constrained | | Model 2C:  Random effects and residual variance classes constrained | |
|  |  |  | Class | Bias int | Bias slope | Bias int | Bias slope | Bias int | Bias slope | Bias int | Bias slope | Bias int | Bias slope | Bias int | Bias slope | Bias int | Bias slope |
| 1:3 | 1:3 | 1:3 | 1 | 0.00 | 0.00 | 0.00 | 0.00 | 0.00 | 0.00 | 0.02 | 0.00 | 0.00 | 0.00 | 0.00 | 0.00 | 0.23 | 0.10 |
|  |  |  | 2 | 0.00 | 0.00 | 0.01 | 0.02 | 0.00 | 0.00 | 0.02 | 0.01 | 0.00 | 0.00 | 0.01 | 0.04 | 0.17 | 0.17 |
|  |  |  | 3 | 0.00 | 0.00 | 0.00 | 0.00 | 0.00 | 0.00 | 0.01 | 0.02 | 0.00 | 0.00 | 0.00 | 0.07 | 0.01 | 0.29 |
| 1:10 | 1:3 | 1:3 | 1 | 0.00 | 0.00 | 0.01 | 0.00 | 0.00 | 0.00 | 0.02 | 0.00 | 0.00 | 0.00 | 0.00 | 0.00 | 0.08 | 0.03 |
|  |  |  | 2 | 0.00 | 0.00 | 0.02 | 0.05 | 0.00 | 0.00 | 0.01 | 0.00 | 0.00 | 0.00 | 0.00 | 0.05 | 0.05 | 0.02 |
|  |  |  | 3 | 0.00 | 0.00 | 0.01 | 0.04 | 0.00 | 0.00 | 0.01 | 0.02 | 0.00 | 0.01 | 0.00 | 0.09 | 0.01 | 0.08 |
| 1:3 | 1:10 | 1:3 | 1 | 0.00 | 0.00 | 0.00 | 0.00 | 0.00 | 0.00 | 0.01 | 0.00 | 0.00 | 0.00 | 0.00 | 0.00 | - | - |
|  |  |  | 2 | 0.00 | 0.00 | 0.01 | 0.01 | 0.00 | 0.00 | 0.01 | 0.06 | 0.00 | 0.00 | 0.01 | 0.00 | - | - |
|  |  |  | 3 | 0.00 | 0.00 | 0.01 | 0.03 | 0.00 | 0.00 | 0.02 | 0.04 | 0.00 | 0.00 | 0.01 | 0.10 | - | - |
| 1:3 | 1:3 | 1:10 | 1 | 0.00 | 0.00 | 0.00 | 0.00 | 0.00 | 0.00 | 0.03 | 0.00 | 0.00 | 0.00 | 0.00 | 0.00 | - | - |
|  |  |  | 2 | 0.00 | 0.00 | 0.01 | 0.01 | 0.00 | 0.00 | 0.02 | 0.04 | 0.00 | 0.00 | 0.01 | 0.02 | - | - |
|  |  |  | 3 | 0.00 | 0.01 | 0.03 | 0.14 | 0.00 | 0.01 | 0.02 | 0.09 | 0.00 | 0.01 | 0.01 | 0.15 | - | - |
| 1:10 | 1:10 | 1:3 | 1 | 0.00 | 0.00 | 0.00 | 0.00 | 0.00 | 0.00 | 0.00 | 0.00 | 0.00 | 0.00 | 0.00 | 0.00 | - | - |
|  |  |  | 2 | 0.00 | 0.00 | 0.00 | 0.01 | 0.00 | 0.00 | 0.00 | 0.01 | 0.00 | 0.00 | 0.01 | 0.07 | - | - |
|  |  |  | 3 | 0.00 | 0.00 | 0.00 | 0.02 | 0.00 | 0.00 | 0.00 | 0.00 | 0.00 | 0.00 | 0.00 | 0.09 | - | - |
| 1:3 | 1:10 | 1:10 | 1 | 0.00 | 0.00 | 0.00 | 0.00 | 0.00 | 0.00 | 0.00 | 0.00 | 0.00 | 0.00 | 0.00 | 0.00 | - | - |
|  |  |  | 2 | 0.00 | 0.00 | 0.00 | 0.01 | 0.00 | 0.00 | 0.01 | 0.03 | 0.00 | 0.00 | 0.01 | 0.08 | - | - |
|  |  |  | 3 | 0.00 | 0.00 | 0.00 | 0.03 | 0.00 | 0.00 | 0.01 | 0.03 | 0.00 | 0.00 | 0.00 | 0.15 | - | - |
| 1:10 | 1:10 | 1:3 | 1 | 0.00 | 0.00 | 0.00 | 0.00 | 0.00 | 0.00 | 0.00 | 0.00 | 0.00 | 0.00 | 0.00 | 0.00 | 0.07 | 0.04 |
|  |  |  | 2 | 0.00 | 0.00 | 0.00 | 0.02 | 0.00 | 0.00 | 0.00 | 0.02 | 0.00 | 0.01 | 0.02 | 0.15 | 0.06 | 0.04 |
|  |  |  | 3 | 0.00 | 0.00 | 0.01 | 0.04 | 0.00 | 0.00 | 0.01 | 0.02 | 0.00 | 0.01 | 0.02 | 0.16 | 0.01 | 0.16 |
| 1:10 | 1:10 | 1:10 | 1 | 0.00 | 0.00 | 0.00 | 0.00 | 0.00 | 0.00 | 0.00 | 0.00 | 0.00 | 0.00 | 0.00 | 0.00 | - | - |
|  |  |  | 2 | 0.00 | 0.00 | 0.00 | 0.02 | 0.00 | 0.00 | 0.00 | 0.05 | 0.00 | 0.00 | 0.02 | 0.19 | - | - |
|  |  |  | 3 | 0.00 | 0.01 | 0.02 | 0.09 | 0.00 | 0.00 | 0.01 | 0.04 | 0.00 | 0.01 | 0.01 | 0.26 | - | - |
| Color codes for relative bias: green < .1; yellow, ≥ .1 and < 2; red, ≥ .2. 1:3 means that the variance in the first class or first time point is three times smaller than in the last class or last time point. 1:10 means that this ratio is 1:10. | | | | | | | | | | | | | | | | | |

| Supplementary Table 3B. Absolute value of relative bias of intercept (int) and slope per simulated scenario and analysis model: findings over the 1000 replications in the simulation for a sample size of 1000 and a low degree of separation between classes. | | | | | | | | | | | | | | | | | |
| --- | --- | --- | --- | --- | --- | --- | --- | --- | --- | --- | --- | --- | --- | --- | --- | --- | --- |
| Residual variance across classes | Residual variance over time | Random effect variances across classes |  | Model 0:  Nothing constrained | | Model 1A:  Residual variance time constrained | | Model 1B:  Residual variance classes constrained | | Model 1C:  Random effects constrained | | Model 2A:  Residual variance time and classes constrained | | Model 2B:  Random effects and residual variance time constrained | | Model 2C:  Random effects and residual variance classes constrained | |
|  |  |  | Class | Bias int | Bias slope | Bias int | Bias slope | Bias int | Bias slope | Bias int | Bias slope | Bias int | Bias slope | Bias int | Bias slope | Bias int | Bias slope |
| 1:3 | 1:3 | 1:3 | 1 | 0.00 | 0.00 | 0.00 | 0.00 | 0.00 | 0.00 | 0.01 | 0.01 | 0.00 | 0.00 | 0.01 | 0.01 | - | - |
|  |  |  | 2 | 0.00 | 0.01 | 0.01 | 0.08 | 0.01 | 0.04 | 0.01 | 0.01 | 0.01 | 0.06 | 0.01 | 0.03 | - | - |
|  |  |  | 3 | 0.00 | 0.01 | 0.00 | 0.08 | 0.00 | 0.00 | 0.02 | 0.05 | 0.00 | 0.00 | 0.01 | 0.21 | - | - |
| 1:10 | 1:3 | 1:3 | 1 | 0.00 | 0.00 | 0.00 | 0.01 | 0.00 | 0.00 | 0.03 | 0.01 | 0.00 | 0.00 | 0.02 | 0.02 | - | - |
|  |  |  | 2 | 0.00 | 0.00 | 0.02 | 0.37 | 0.00 | 0.01 | 0.00 | 0.02 | 0.02 | 0.00 | 0.02 | 0.06 | - | - |
|  |  |  | 3 | 0.00 | 0.00 | 0.02 | 0.28 | 0.00 | 0.00 | 0.01 | 0.06 | 0.00 | 0.01 | 0.01 | 0.34 | - | - |
| 1:3 | 1:10 | 1:3 | 1 | 0.00 | 0.00 | 0.00 | 0.00 | 0.00 | 0.00 | 0.01 | 0.01 | 0.00 | 0.00 | 0.01 | 0.01 | - | - |
|  |  |  | 2 | 0.00 | 0.00 | 0.01 | 0.07 | 0.00 | 0.00 | 0.00 | 0.18 | 0.00 | 0.01 | 0.01 | 0.07 | - | - |
|  |  |  | 3 | 0.00 | 0.01 | 0.01 | 0.10 | 0.00 | 0.01 | 0.06 | 0.12 | 0.00 | 0.02 | 0.03 | 0.28 | - | - |
| 1:3 | 1:3 | 1:10 | 1 | 0.00 | 0.00 | 0.00 | 0.00 | 0.00 | 0.00 | 0.04 | 0.01 | 0.00 | 0.00 | 0.01 | 0.02 | - | - |
|  |  |  | 2 | 0.00 | 0.00 | 0.03 | 0.21 | 0.00 | 0.00 | 0.01 | 0.08 | 0.00 | 0.00 | 0.00 | 0.07 | - | - |
|  |  |  | 3 | 0.00 | 0.01 | 0.02 | 0.26 | 0.00 | 0.01 | 0.03 | 0.20 | 0.00 | 0.02 | 0.02 | 0.39 | - | - |
| 1:10 | 1:10 | 1:3 | 1 | 0.00 | 0.00 | 0.00 | 0.00 | 0.00 | 0.00 | 0.00 | 0.00 | 0.00 | 0.00 | 0.00 | 0.00 | - | - |
|  |  |  | 2 | 0.00 | 0.00 | 0.00 | 0.04 | 0.01 | 0.02 | 0.01 | 0.02 | 0.01 | 0.01 | 0.02 | 0.11 | - | - |
|  |  |  | 3 | 0.00 | 0.00 | 0.00 | 0.05 | 0.00 | 0.01 | 0.01 | 0.01 | 0.00 | 0.01 | 0.00 | 0.22 | - | - |
| 1:3 | 1:10 | 1:10 | 1 | 0.00 | 0.00 | 0.00 | 0.00 | 0.00 | 0.00 | 0.00 | 0.00 | 0.00 | 0.00 | 0.00 | 0.00 | - | - |
|  |  |  | 2 | 0.00 | 0.00 | 0.00 | 0.04 | 0.00 | 0.00 | 0.02 | 0.08 | 0.00 | 0.01 | 0.01 | 0.11 | - | - |
|  |  |  | 3 | 0.00 | 0.01 | 0.00 | 0.05 | 0.00 | 0.02 | 0.04 | 0.09 | 0.00 | 0.03 | 0.01 | 0.35 | - | - |
| 1:10 | 1:10 | 1:3 | 1 | 0.00 | 0.00 | 0.00 | 0.00 | 0.00 | 0.00 | 0.00 | 0.00 | 0.00 | 0.00 | 0.00 | 0.00 | - | - |
|  |  |  | 2 | 0.00 | 0.00 | 0.01 | 0.14 | 0.00 | 0.01 | 0.01 | 0.04 | 0.00 | 0.01 | 0.03 | 0.19 | - | - |
|  |  |  | 3 | 0.00 | 0.01 | 0.01 | 0.10 | 0.00 | 0.01 | 0.01 | 0.02 | 0.00 | 0.02 | 0.02 | 0.35 | - | - |
| 1:10 | 1:10 | 1:10 | 1 | 0.00 | 0.00 | 0.00 | 0.00 | 0.00 | 0.00 | 0.00 | 0.00 | 0.00 | 0.00 | 0.00 | 0.00 | - | - |
|  |  |  | 2 | 0.00 | 0.00 | 0.00 | 0.11 | 0.00 | 0.00 | 0.01 | 0.11 | 0.00 | 0.00 | 0.03 | 0.24 | - | - |
|  |  |  | 3 | 0.00 | 0.01 | 0.01 | 0.13 | 0.00 | 0.02 | 0.03 | 0.07 | 0.00 | 0.03 | 0.01 | 0.53 | - | - |
| Color codes for relative bias: green < .1; yellow, ≥ .1 and < 2; red, ≥ .2  1:3 means that the variance in the first class or first time point is three times smaller than in the last class or last time point. 1:10 means that this ratio is 1:10. | | | | | | | | | | | | | | | | | |

| Supplementary Table 3C. Absolute value of relative bias of intercept (int) and slope per simulated scenario and analysis model: findings over the 1000 replications in the simulation for a sample size of 300 and a low degree of separation between classes. | | | | | | | | | | | | | | | | | | |  |
| --- | --- | --- | --- | --- | --- | --- | --- | --- | --- | --- | --- | --- | --- | --- | --- | --- | --- | --- | --- |
| Residual variance across classes | Residual variance over time | Random effect variances across classes |  | Model 0:  Nothing constrained | | Model 1A:  Residual variance time constrained | | Model 1B:  Residual variance classes constrained | | Model 1C:  Random effects constrained | | Model 2A:  Residual variance time and classes constrained | | Model 2B:  Random effects and residual variance time constrained | | Model 2C:  Random effects and residual variance classes constrained | | |  |
|  |  |  | Class | Bias int | Bias slope | Bias int | Bias slope | Bias int | Bias slope | Bias int | Bias slope | Bias int | Bias slope | Bias int | Bias slope | Bias int | Bias slope | |  |
| 1:3 | 1:3 | 1:3 | 1 | 0.00 | 0.00 | 0.00 | 0.00 | 0.01 | 0.00 | 0.01 | 0.01 | 0.01 | 0.00 | 0.01 | 0.01 | 0.13 | 0.08 | |  |
|  |  |  | 2 | 0.01 | 0.03 | 0.01 | 0.10 | 0.03 | 0.10 | 0.01 | 0.00 | 0.04 | 0.22 | 0.02 | 0.03 | 0.29 | 1.23 | |  |
|  |  |  | 3 | 0.00 | 0.01 | 0.00 | 0.08 | 0.00 | 0.00 | 0.02 | 0.04 | 0.00 | 0.03 | 0.01 | 0.20 | 0.03 | 0.37 | |  |
| 1:10 | 1:3 | 1:3 | 1 | 0.00 | 0.00 | 0.00 | 0.01 | 0.00 | 0.00 | 0.03 | 0.01 | 0.01 | 0.01 | 0.02 | 0.02 | 0.13 | 0.12 | |  |
|  |  |  | 2 | 0.00 | 0.01 | 0.01 | 0.34 | 0.01 | 0.03 | 0.00 | 0.03 | 0.04 | 0.02 | 0.03 | 0.08 | 0.15 | 0.55 | |  |
|  |  |  | 3 | 0.00 | 0.01 | 0.02 | 0.22 | 0.00 | 0.02 | 0.01 | 0.06 | 0.00 | 0.03 | 0.01 | 0.40 | 0.02 | 0.29 | |  |
| 1:3 | 1:10 | 1:3 | 1 | 0.00 | 0.00 | 0.00 | 0.00 | 0.00 | 0.00 | 0.01 | 0.01 | 0.00 | 0.00 | 0.01 | 0.02 | - | - | |  |
|  |  |  | 2 | 0.00 | 0.01 | 0.01 | 0.07 | 0.01 | 0.00 | 0.01 | 0.19 | 0.01 | 0.01 | 0.01 | 0.08 | - | - | |  |
|  |  |  | 3 | 0.00 | 0.02 | 0.00 | 0.07 | 0.01 | 0.02 | 0.07 | 0.12 | 0.01 | 0.02 | 0.03 | 0.30 | - | - | |  |
| 1:3 | 1:3 | 1:10 | 1 | 0.00 | 0.00 | 0.00 | 0.00 | 0.00 | 0.00 | 0.04 | 0.02 | 0.00 | 0.00 | 0.01 | 0.02 | 0.17 | 0.11 | |  |
|  |  |  | 2 | 0.00 | 0.01 | 0.02 | 0.23 | 0.01 | 0.02 | 0.00 | 0.12 | 0.01 | 0.01 | 0.01 | 0.06 | 0.13 | 0.04 | |  |
|  |  |  | 3 | 0.00 | 0.02 | 0.01 | 0.23 | 0.01 | 0.03 | 0.05 | 0.16 | 0.01 | 0.04 | 0.02 | 0.42 | 0.07 | 0.52 | |  |
| 1:10 | 1:10 | 1:3 | 1 | 0.00 | 0.00 | 0.00 | 0.00 | 0.00 | 0.00 | 0.00 | 0.00 | 0.00 | 0.00 | 0.00 | 0.00 | 0.19 | 0.05 | |  |
|  |  |  | 2 | 0.00 | 0.01 | 0.00 | 0.04 | 0.02 | 0.08 | 0.01 | 0.01 | 0.02 | 0.08 | 0.02 | 0.12 | 0.22 | 1.41 | |  |
|  |  |  | 3 | 0.00 | 0.01 | 0.00 | 0.03 | 0.00 | 0.04 | 0.02 | 0.01 | 0.00 | 0.03 | 0.00 | 0.23 | 0.04 | 1.29 | |  |
| 1:3 | 1:10 | 1:10 | 1 | 0.00 | 0.00 | 0.00 | 0.00 | 0.00 | 0.00 | 0.00 | 0.00 | 0.00 | 0.00 | 0.00 | 0.00 | 0.18 | 0.01 | |  |
|  |  |  | 2 | 0.00 | 0.00 | 0.00 | 0.04 | 0.01 | 0.00 | 0.02 | 0.08 | 0.00 | 0.01 | 0.01 | 0.11 | 0.45 | 0.71 | |  |
|  |  |  | 3 | 0.00 | 0.03 | 0.00 | 0.04 | 0.00 | 0.06 | 0.04 | 0.10 | 0.00 | 0.06 | 0.01 | 0.36 | 0.21 | 2.82 | |  |
| 1:10 | 1:10 | 1:3 | 1 | 0.00 | 0.00 | 0.00 | 0.00 | 0.00 | 0.00 | 0.00 | 0.00 | 0.00 | 0.00 | 0.00 | 0.00 | 0.15 | 0.05 | |  |
|  |  |  | 2 | 0.00 | 0.01 | 0.01 | 0.16 | 0.01 | 0.02 | 0.01 | 0.04 | 0.02 | 0.06 | 0.04 | 0.20 | 0.22 | 0.14 | |  |
|  |  |  | 3 | 0.00 | 0.01 | 0.01 | 0.11 | 0.00 | 0.04 | 0.01 | 0.01 | 0.00 | 0.03 | 0.02 | 0.38 | 0.05 | 1.27 | |  |
| 1:10 | 1:10 | 1:10 | 1 | 0.00 | 0.00 | 0.00 | 0.00 | 0.00 | 0.00 | 0.00 | 0.00 | 0.00 | 0.00 | 0.00 | 0.00 | - | - | |  |
|  |  |  | 2 | 0.00 | 0.00 | 0.00 | 0.13 | 0.00 | 0.00 | 0.01 | 0.13 | 0.00 | 0.01 | 0.03 | 0.22 | - | - | |  |
|  |  |  | 3 | 0.00 | 0.00 | 0.01 | 0.13 | 0.00 | 0.03 | 0.04 | 0.06 | 0.01 | 0.06 | 0.01 | 0.53 | - | - | |  |
| Color codes for relative bias: green < .1; yellow, ≥ .1 and < 2; red, ≥ .2 1:3 means that the variance in the first class or first time point is three times smaller than in the last class or last time point. 1:10 means that this ratio is 1:10. | | | | | | | | | | | | | | | | | | |  |
| Supplementary Table 3D Absolute value of relative bias of intercept (int) and slope per simulated scenario and analysis model: findings over the 1000 replications in the simulation for a sample size of 100 and a low degree of separation between classes. | | | | | | | | | | | | | | | | | | | |
| Residual variance classes | Residual variance time | Random effect variances |  | Model 0:  Nothing constrained | | Model 1A:  Residual variance time constrained | | Model 1B:  Residual variance classes constrained | | Model 1C:  Random effects constrained | | Model 2A:  Residual variance time and classes constrained | | Model 2B:  Random effects and residual variance time constrained | | Model 2C:  Random effects and residual variance classes constrained | | | |
|  |  |  | Class | Bias int | Bias slope | Bias int | Bias slope | Bias int | Bias slope | Bias int | Bias slope | Bias int | Bias slope | Bias int | Bias slope | Bias int | | Bias slope | |
| 1:3 | 1:3 | 1:3 | 1 | 0.01 | 0.01 | 0.01 | 0.01 | 0.01 | 0.00 | 0.02 | 0.01 | 0.02 | 0.02 | 0.01 | 0.01 | 0.13 | | 0.03 | |
|  |  |  | 2 | 0.05 | 0.04 | 0.02 | 0.08 | 0.05 | 0.07 | 0.02 | 0.06 | 0.07 | 0.37 | 0.03 | 0.08 | 0.19 | | 1.06 | |
|  |  |  | 3 | 0.01 | 0.18 | 0.01 | 0.00 | 0.01 | 0.18 | 0.02 | 0.07 | 0.01 | 0.02 | 0.01 | 0.26 | 0.01 | | 0.47 | |
| 1:10 | 1:3 | 1:3 | 1 | 0.00 | 0.00 | 0.01 | 0.01 | 0.01 | 0.01 | 0.03 | 0.01 | 0.02 | 0.01 | 0.02 | 0.02 | 0.12 | | 0.06 | |
|  |  |  | 2 | 0.03 | 0.12 | 0.02 | 0.15 | 0.04 | 0.04 | 0.01 | 0.01 | 0.08 | 0.04 | 0.04 | 0.10 | 0.16 | | 0.53 | |
|  |  |  | 3 | 0.01 | 0.16 | 0.02 | 0.04 | 0.01 | 0.12 | 0.02 | 0.09 | 0.02 | 0.03 | 0.01 | 0.48 | 0.02 | | 0.41 | |
| 1:3 | 1:10 | 1:3 | 1 | 0.00 | 0.00 | 0.00 | 0.00 | 0.00 | 0.01 | 0.02 | 0.02 | 0.01 | 0.01 | 0.01 | 0.02 | 0.14 | | 0.03 | |
|  |  |  | 2 | 0.04 | 0.03 | 0.01 | 0.04 | 0.04 | 0.11 | 0.02 | 0.16 | 0.04 | 0.09 | 0.02 | 0.07 | 0.20 | | 0.88 | |
|  |  |  | 3 | 0.01 | 0.20 | 0.00 | 0.01 | 0.00 | 0.21 | 0.07 | 0.14 | 0.01 | 0.04 | 0.04 | 0.33 | 0.05 | | 0.76 | |
| 1:3 | 1:3 | 1:10 | 1 | 0.00 | 0.00 | 0.00 | 0.00 | 0.00 | 0.00 | 0.04 | 0.02 | 0.00 | 0.00 | 0.01 | 0.02 | 0.10 | | 0.01 | |
|  |  |  | 2 | 0.02 | 0.09 | 0.00 | 0.25 | 0.02 | 0.10 | 0.01 | 0.18 | 0.04 | 0.11 | 0.01 | 0.06 | 0.13 | | 0.03 | |
|  |  |  | 3 | 0.00 | 0.11 | 0.01 | 0.08 | 0.01 | 0.08 | 0.06 | 0.13 | 0.01 | 0.06 | 0.03 | 0.48 | 0.14 | | 0.18 | |
| 1:10 | 1:10 | 1:3 | 1 | 0.00 | 0.00 | 0.00 | 0.00 | 0.01 | 0.00 | 0.00 | 0.00 | 0.01 | 0.00 | 0.00 | 0.00 | 0.19 | | 0.02 | |
|  |  |  | 2 | 0.01 | 0.03 | 0.01 | 0.06 | 0.06 | 0.02 | 0.01 | 0.01 | 0.04 | 0.16 | 0.02 | 0.13 | 0.24 | | 1.27 | |
|  |  |  | 3 | 0.01 | 0.14 | 0.01 | 0.03 | 0.02 | 0.34 | 0.01 | 0.06 | 0.01 | 0.12 | 0.01 | 0.29 | 0.10 | | 1.81 | |
| 1:3 | 1:10 | 1:10 | 1 | 0.00 | 0.00 | 0.00 | 0.00 | 0.00 | 0.00 | 0.00 | 0.00 | 0.00 | 0.00 | 0.00 | 0.00 | 0.17 | | 0.03 | |
|  |  |  | 2 | 0.00 | 0.02 | 0.00 | 0.05 | 0.04 | 0.05 | 0.02 | 0.07 | 0.03 | 0.01 | 0.01 | 0.13 | 0.25 | | 1.04 | |
|  |  |  | 3 | 0.00 | 0.12 | 0.00 | 0.02 | 0.00 | 0.31 | 0.04 | 0.12 | 0.00 | 0.26 | 0.01 | 0.40 | 0.00 | | 1.22 | |
| 1:10 | 1:10 | 1:3 | 1 | 0.00 | 0.00 | 0.00 | 0.00 | 0.01 | 0.01 | 0.00 | 0.00 | 0.01 | 0.00 | 0.00 | 0.00 | 0.14 | | 0.04 | |
|  |  |  | 2 | 0.01 | 0.03 | 0.02 | 0.21 | 0.04 | 0.04 | 0.00 | 0.05 | 0.05 | 0.28 | 0.04 | 0.19 | 0.17 | | 0.55 | |
|  |  |  | 3 | 0.00 | 0.07 | 0.01 | 0.06 | 0.01 | 0.23 | 0.02 | 0.04 | 0.01 | 0.10 | 0.02 | 0.53 | 0.02 | | 1.07 | |
| 1:10 | 1:10 | 1:10 | 1 | 0.00 | 0.00 | 0.00 | 0.00 | 0.00 | 0.00 | 0.00 | 0.00 | 0.00 | 0.00 | 0.00 | 0.00 | 0.16 | | 0.04 | |
|  |  |  | 2 | 0.00 | 0.03 | 0.01 | 0.17 | 0.03 | 0.09 | 0.01 | 0.14 | 0.03 | 0.01 | 0.03 | 0.24 | 0.17 | | 0.63 | |
|  |  |  | 3 | 0.00 | 0.08 | 0.01 | 0.06 | 0.00 | 0.16 | 0.05 | 0.07 | 0.00 | 0.38 | 0.01 | 0.59 | 0.03 | | 0.69 | |
| Color codes for relative bias: green < .1; yellow, ≥ .1 and < 2; red, ≥ .2  1:3 means that the variance in the first class or first time point is three times smaller than in the last class or last time point. 1:10 means that this ratio is 1:10. | | | | | | | | | | | | | | | | | | | |

| Supplementary Table 4. Model fit Indices for growth mixture models of aggressive behavior in the TRacking Adolescent Individuals’ Lives Survey (TRAILS), The Netherlands, 2001-2017, by model | | | | | | | | | | | | |
| --- | --- | --- | --- | --- | --- | --- | --- | --- | --- | --- | --- | --- |
| Model | # Classes | BIC | aBIC | AIC | Entropy | LMR-LRT | VLMR-LRT | Class 1 | Class 2 | Class 3 | Class 4 | Class 5 |
| 0: Unconstrained | 1^a^ | -2326 | -2367 | -2400 | - | - | - | - | - | - | - | - |
|  | 2 | -4393 | -4475 | -4541 | 0.69 | 0.00 | 0.00 | 0.60 | 0.40 | - | - | - |
|  | 3 | -4885 | -5009 | -5107 | 0.67 | 0.01 | 0.01 | 0.17 | 0.36 | 0.47 | - | - |
| 1A: Residual variance time constrained | 1^b^ | -2300 | -2325 | -2345 | - | - | - | - | - | - | - | - |
|  | 2 | -4090 | -4141 | -4182 | 0.70 | 0.00 | 0.00 | 0.39 | 0.61 | - | - | - |
|  | 3 | -4553 | -4630 | -4690 | 0.65 | 0.00 | 0.00 | 0.46 | 0.22 | 0.33 | - | - |
|  | 4 | -4738 | -4840 | -4921 | 0.66 | 0.01 | 0.01 | 0.41 | 0.08 | 0.25 | 0.27 | - |
|  | 5 | -4929 | -5056 | -5157 | 0.68 | 0.03 | 0.03 | 0.25 | 0.36 | 0.08 | 0.23 | 0.09 |
| 1B: Residual variance classes constrained | 2 | -3421 | -3506 | -3575 | 0.56 | 0.00 | 0.00 | 0.54 | 0.46 | - | - | - |
|  | 3 | -3149 | -3235 | -3303 | 0.54 | 0.13 | 0.13 | 0.52 | 0.34 | 0.14 | - | - |
|  | 4 | -3261 | -3369 | -3455 | 0.54 | 0.05 | 0.05 | 0.33 | 0.13 | 0.14 | 0.40 | - |
| 1C: Random effects constrained | 2 | -4143 | -4219 | -4280 | 0.67 | 0.00 | 0.00 | 0.51 | 0.49 | - | - | - |
|  | 3 | -4791 | -4902 | -4991 | 0.66 | 0.03 | 0.03 | 0.44 | 0.24 | 0.32 | - | - |
|  |  |  |  |  |  |  |  |  |  |  |  |  |
| 2A: Residual variance constrained  time and classes | 2 | -2873 | -2921 | -2959 | 0.46 | 0.00 | 0.00 | 0.54 | 0.46 | - | - | - |
|  | 3 | -3029 | -3099 | -3154 | 0.58 | 0.00 | 0.00 | 0.42 | 0.11 | 0.47 | - | - |
|  | 4 | -3186 | -3278 | -3352 | 0.55 | 0.19 | 0.18 | 0.43 | 0.31 | 0.13 | 0.14 | - |
|  | 5 | -3360 | -3474 | -3565 | 0.59 | 0.01 | 0.01 | 0.37 | 0.32 | 0.12 | 0.09 | 0.10 |
| 2B: Random effects and  residual variance time constrained | 1 | -2300 | -2325 | -2345 | - | - | - | - | - | - | - | - |
|  | 2 | -3843 | -3888 | -3923 | 0.66 | 0.00 | 0.00 | 0.47 | 0.53 | - | - | - |
|  | 3 | -4459 | -4522 | -4573 | 0.70 | 0.00 | 0.00 | 0.51 | 0.26 | 0.23 | - | - |
|  | 4 | -4685 | -4768 | -4833 | 0.71 | 0.00 | 0.00 | 0.26 | 0.09 | 0.43 | 0.22 | - |
|  | 5 | -4889 | -4991 | -5072 | 0.71 | 0.00 | 0.00 | 0.09 | 0.08 | 0.39 | 0.24 | 0.20 |
| 2C: Random effects and  residual variance classes constrained |  |  |  |  |  |  |  |  |  |  |  |  |
|  | 2 | -2705 | -2763 | -2808 | 0.79 | 0.00 | 0.00 | 0.86 | 0.14 | - | - | - |
|  | 3 | -2916 | -2989 | -3047 | 0.78 | 0.00 | 0.00 | 0.09 | 0.79 | 0.11 | - | - |
|  | 4 | -3032 | -3121 | -3192 | 0.76 | 0.01 | 0.01 | 0.09 | 0.11 | 0.09 | 0.71 | - |
|  | 5 | -3155 | -3260 | -3344 | 0.73 | 0.00 | 0.00 | 0.66 | 0.05 | 0.13 | 0.06 | 0.10 |
| For some models, solutions are not shown for all number of classes because in those instances no solutions could be computed. . Model 0 and 1C: 4 and 5 classes, model 1B: 5 classes. a) This model is the same as the 1-class models 1B, 1C and 2C, b) This model is the same as the 1-class models 2A and 2B. BIC: Bayesian Information Criterion, aBIC: adjusted BIC, AIC: Aikake Information Criterion, LMR-LRT: Lo-Mendell-Rubin Likelihood Ratio Test, VLMR-LRT: Vuong-Lo-Mendell-Rubin LRT | | | | | | | | | | | | |

**Supplementary Figure 1**: Line plots for the first of the 1000 datasets in the simulated data in scenario 1.


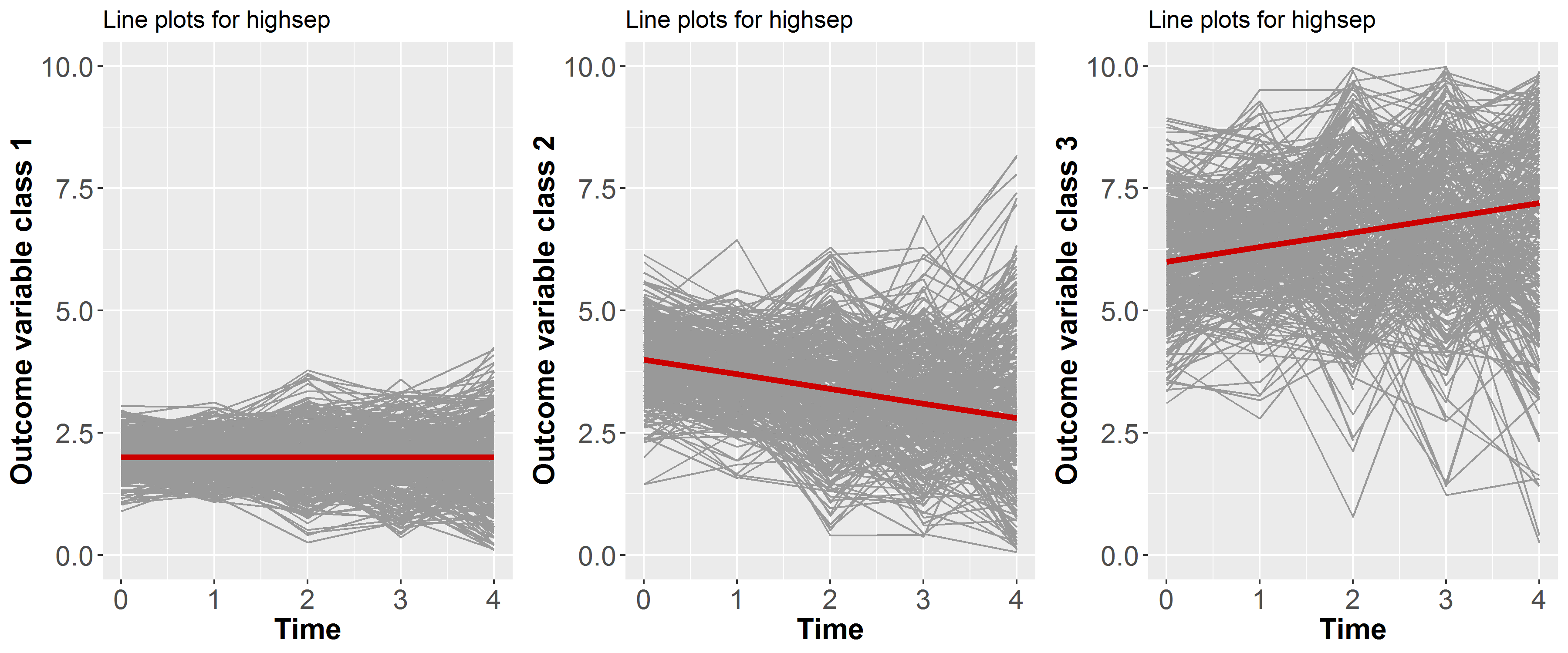


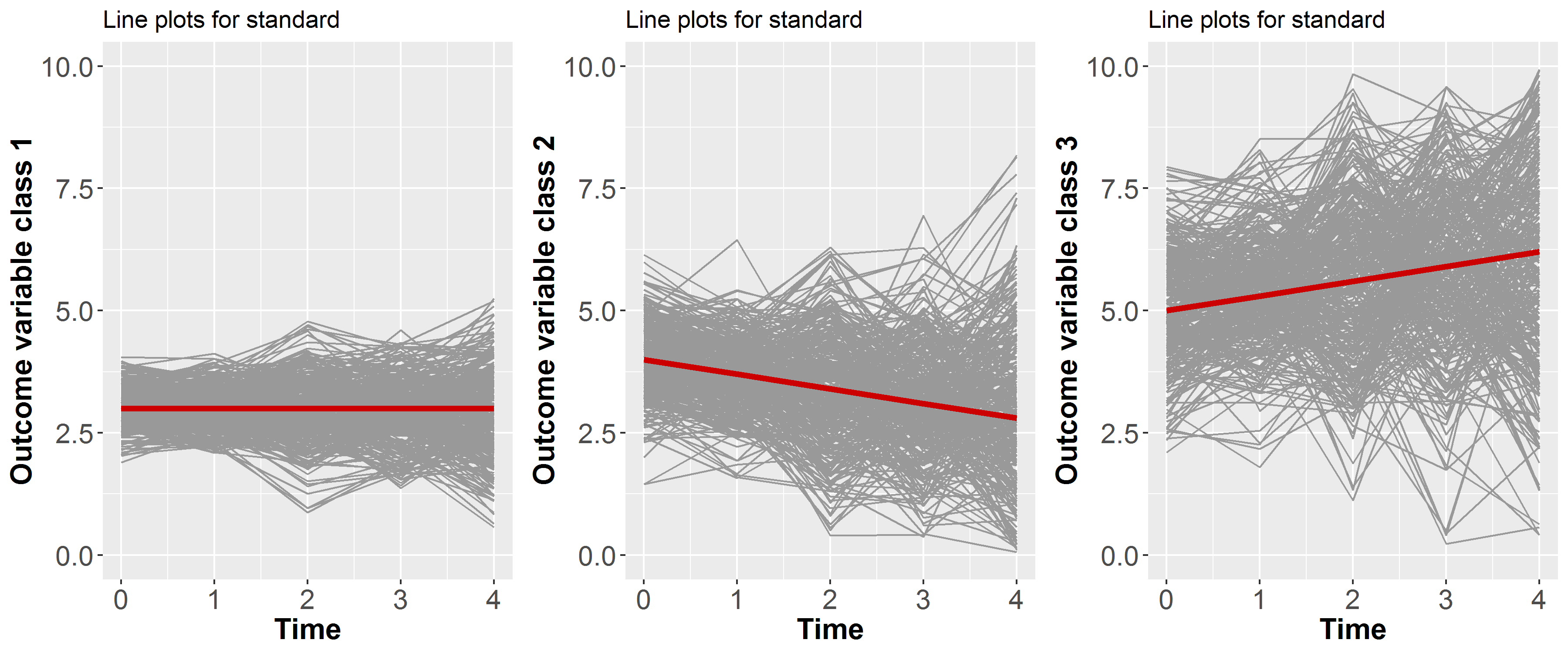


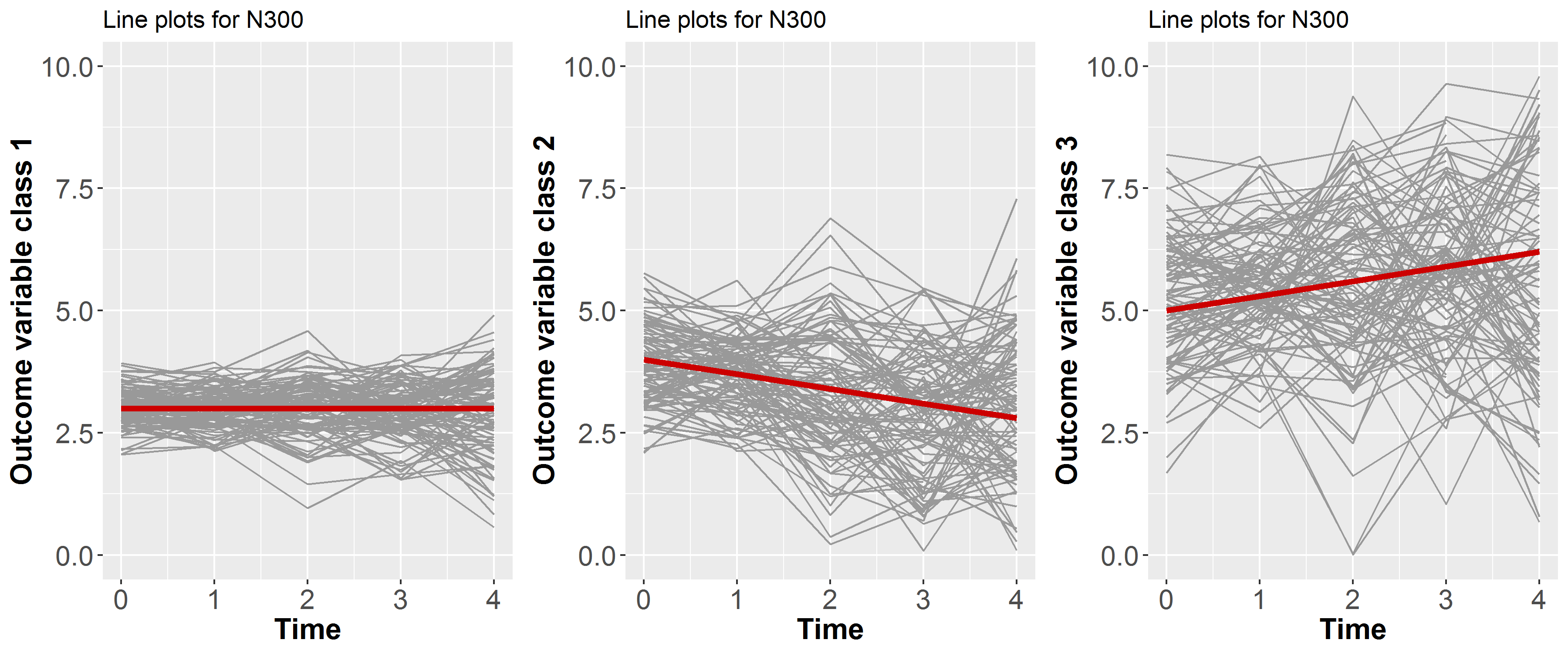


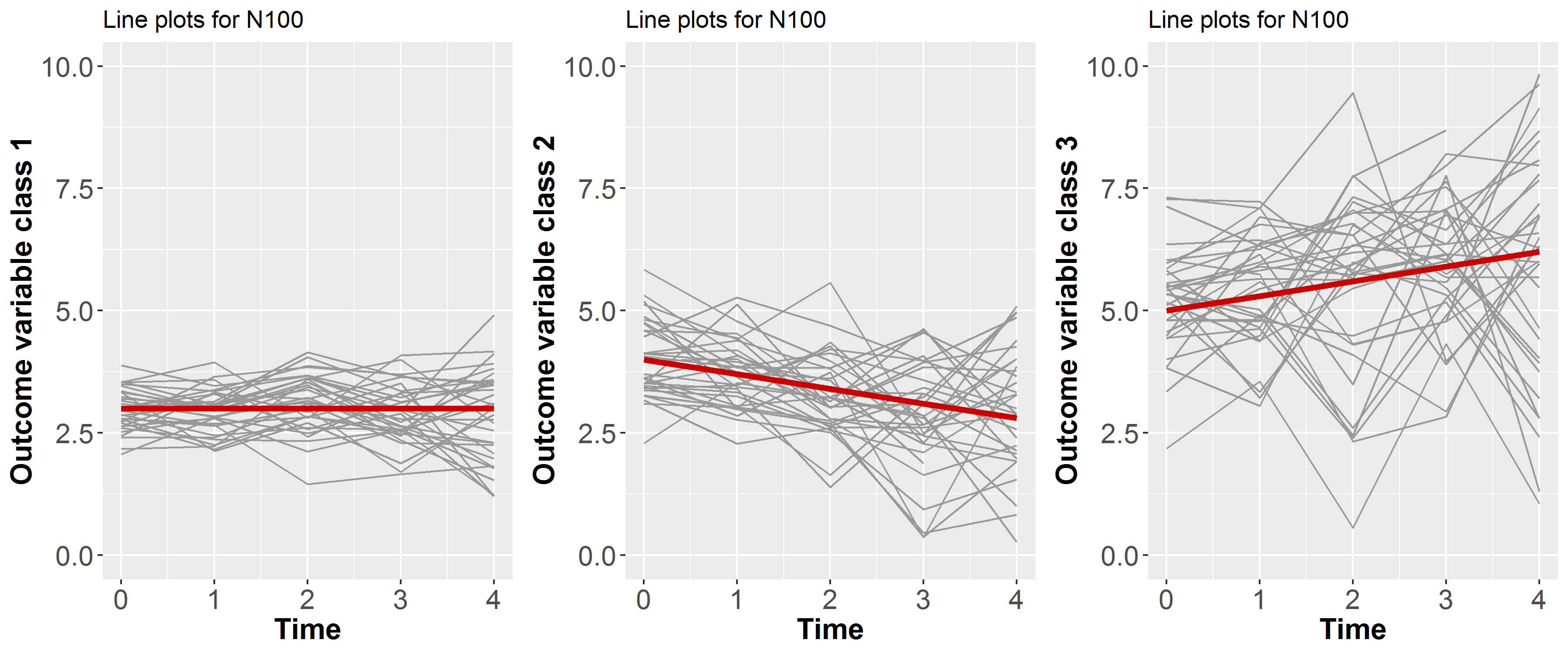

Supplement: Supplementary file 1 — Additional file 1: Table S1. How often was each model selected to be best fitting according to several fit indices in the simulation study: text bolded for the lowest (information criteria) or highest (entropy) value. Table S2. Class recovery, occurrence of negative variances and individuals correctly classified over 1000 replications, by sample sizes (N), degrees of separation, and ratios of residual variances (var.) and random effects in the data generation process. Table S3A. Absolute value of relative bias of intercept (int) and slope per simulated scenario and analysis model: findings over the 1000 replications in the simulation for a sample size of 1000 and a high degree of separation between classes. Table S3B. Absolute value of relative bias of intercept (int) and slope per simulated scenario and analysis model: findings over the 1000 replications in the simulation for a sample size of 1000 and a low degree of separation between classes. Table S3C. Absolute value of relative bias of intercept (int) and slope per simulated scenario and analysis model: findings over the 1000 replications in the simulation for a sample size of 300 and a low degree of separation between classes. Table S3D. Absolute value of relative bias of intercept (int) and slope per simulated scenario and analysis model: findings over the 1000 replications in the simulation for a sample size of 100 and a low degree of separation between classes. Table S4. Model fit Indices for growth mixture models of aggressive behavior in the TRacking Adolescent Individuals’ Lives Survey (TRAILS), The Netherlands, 2001–2017, by model. Figure S1. Line plots for the first of the 1000 datasets in the simulated data in scenario 1. [file 12874_2020_1154_MOESM1_ESM.docx]
